# Integrating multiple data sources (MUDS) for meta-analysis to improve patient-centered outcomes research: a protocol for a systematic review

**DOI:** 10.1186/s13643-015-0134-z

**Published:** 2015-11-02

**Authors:** Evan Mayo-Wilson, Susan Hutfless, Tianjing Li, Gillian Gresham, Nicole Fusco, Jeffrey Ehmsen, James Heyward, Swaroop Vedula, Diana Lock, Jennifer Haythornthwaite, Jennifer L. Payne, Theresa Cowley, Elizabeth Tolbert, Lori Rosman, Claire Twose, Elizabeth A. Stuart, Hwanhee Hong, Peter Doshi, Catalina Suarez-Cuervo, Sonal Singh, Kay Dickersin

**Affiliations:** 1 Center for Clinical Trials and Evidence Synthesis, Department of Epidemiology, Johns Hopkins Bloomberg School of Public Health, 615 North Wolfe Street, Baltimore, MD 21205 USA; 2 Department of Gastroenterology and Hepatology, Johns Hopkins School of Medicine, 600 North Wolfe Street, Blalock 449, Baltimore, MD 21287 USA; 3 Department of Neurology, Johns Hopkins School of Medicine, 855 North Wolfe Street, Baltimore, MD 21205 USA; 4 Laboratory for Computational Sensing and Robotics, Johns Hopkins Whiting School of Engineering, 3400 North Charles St., Baltimore, MD 21218 USA; 5 Department of Health Policy and Management, Johns Hopkins Bloomberg School of Public Health, 615 North Wolfe Street, Baltimore, MD 21205 USA; 6 Behavioral Biology, Center for Mind-Body Research, Johns Hopkins School of Medicine, 5501 Hopkins Bayview Circle, Baltimore, 21224 MD USA; 7 Psychiatry and Behavioral Sciences, Johns Hopkins School of Medicine, 500 North Broadway, Suite 305, Baltimore, MD 21205 USA; 8 The TMJ Association, Ltd., P.O. Box 26770, Milwaukee, WI 53226-0770 USA; 9 Peabody Institute, Johns Hopkins University, 1 East Mount Vernon Place, Baltimore, MD 21202 USA; 10 Welch Medical Library, Johns Hopkins School of Medicine, 615 North Wolfe Street, Baltimore, MD 21205 USA; 11 Department of Mental Health, Johns Hopkins Bloomberg School of Public Health, 624 North Broadway, Baltimore, MD 21205 USA; 12 University of Maryland School of Pharmacy, 220 Arch Street, Baltimore, MD 21201 USA; 13 Center for Public Health and Human Rights, Johns Hopkins School of Medicine, 615 N Wolfe St, Baltimore, MD 21205 USA

**Keywords:** Systematic reviews, Meta-analysis, Reporting bias, Publication bias, Guidance, Gabapentin, Pain, Quetiapine, Depression, Bipolar disorder

## Abstract

**Background:**

Systematic reviews should provide trustworthy guidance to decision-makers, but their credibility is challenged by the selective reporting of trial results and outcomes. Some trials are not published, and even among clinical trials that are published partially (e.g., as conference abstracts), many are never published in full. Although there are many potential sources of published and unpublished data for systematic reviews, there are no established methods for choosing among multiple reports or data sources about the same trial.

**Methods:**

We will conduct systematic reviews of the effectiveness and safety of two interventions following the Institute of Medicine (IOM) guidelines: (1) gabapentin for neuropathic pain and (2) quetiapine for bipolar depression. For the review of gabapentin, we will include adult participants with neuropathic pain who do not require ventilator support. For the review of quetiapine, we will include adult participants with acute bipolar depression (excluding mixed or rapid cycling episodes). We will compare these drugs (used alone or in combination with other interventions) with placebo or with the same intervention alone; direct comparisons with other medications will be excluded. For each review, we will conduct highly sensitive electronic searches, and the results of the searches will be assessed by two independent reviewers. Outcomes, study characteristics, and risk of bias ratings will be extracted from multiple reports by two individuals working independently, stored in a publicly available database (Systematic Review Data Repository) and analyzed using commonly available statistical software. In each review, we will conduct a series of meta-analyses using data from different sources to determine how the results are affected by the inclusion of data from multiple published sources (e.g., journal articles and conference abstracts) as well as unpublished aggregate data (e.g., “clinical study reports”) and individual participant data (IPD). We will identify patient-centered outcomes in each report and identify differences in the reporting of these outcomes across sources.

**Systematic review registration:**

CRD42015014037, CRD42015014038

## Background

### Multiple sources of data

Systematic reviews and meta-analyses are comparative effectiveness research methods that involve summarizing existing research to establish how well treatments work. Systematic reviews should provide trustworthy guidance to decision-makers, but their credibility is challenged by the selective reporting of trial results and outcomes. Some trials are not published, and even among clinical trials that are published partially (e.g., as conference abstracts), many are never published in full [1].

Failure to publish is not random. Studies favoring the comparator or null findings are less likely to be published compared with studies that favor the test treatment, known as *publication bias* [2]. Even when studies are published, authors may report selectively the statistically significant outcomes favoring the test treatment; they may not publish outcomes favoring the comparator or outcomes for which there were no statistical differences observed between treatments. This is known as *selective outcome reporting bias* [3]. Additionally, analyses of reported trials are often not done on an “intention to treat” basis, in which all randomized patients are analyzed as part of the group to which they were assigned, leaving such results vulnerable to selection bias despite randomization [4]. Readers may estimate results for dichotomous outcomes with missing cases by assuming that participants did or did not improve, and systematic reviewers can conduct sensitivity analyses to explore how continuous variables are affected by post-randomization exclusions, but the validity of the assumptions underlying these methods may be difficult to test.

Failure to report research is a tremendous waste [5]. In addition, and of arguably more importance, treatment decisions on the basis of biased reporting may harm people by the prescription of treatments that are less effective and more harmful than what the systematic reviews suggest. Thus, to decrease the threat of reporting biases, current best practices for the conduct of systematic reviews include searching for unpublished trial results as well as the “grey” literature such as conference abstracts [6].

There are many potential sources of published and unpublished data, and there are no established methods for choosing among multiple reports or data sources about the same trial. Moreover, most reports such as journal publications and trial registries include only data summaries at the group level (i.e., aggregate data), and other reports (e.g., conference abstracts, posters, and regulatory packets submitted for approval) may include only fragmentary and incomplete study details. Some sources, such as conference abstracts, may be so selectively reported that their inclusion in a meta-analysis could increase rather than reduce bias [7]; however, supplementing short reports with additional information from trial registries could provide a more complete account of trials when longer reports are unavailable [8]. Individual participant data (IPD), by contrast, may include more details than reports of aggregate results, and IPD can be re-analyzed when patient data have been omitted from published analyses. However, IPD are rarely available for all included studies in systematic reviews.

A few studies have examined possible methods for supplementing information from published reports. For example, studies have compared the reliability of meta-analytic estimates in systematic reviews using data submitted to the Food and Drug Administration (FDA) and published data [9]. There are numerous cases in which unpublished trials have come to light and summary data from those studies have been used in systematic reviews about clinical efficacy, but these cases typically pick a perceived “best” source for each meta-analysis and do not show how meta-analyses would be affected by the inclusion of data from different sources [10–12]. These studies show that when information is present from multiple sources, it does not always agree, and there are no guidelines for choosing which data to include in a systematic review under these circumstances.

IPD are generally considered the best data for performing traditional meta-analysis, not aggregate (summary) data [13]. While authors have noted that “individual participant data are not needed if all the required aggregate data can be obtained in full,” the reality is that reviewers rarely know when this is the case [14]. In addition, analyzing IPD without detailed attention to other elements of study design (such as details about data collection) can lead to superficial and erroneous interpretations of results. One study compared differences between meta-analyses using published data and meta-analyses using IPD [12, 15], and a second meta-analysis using the same data noted that internal correspondence and other documents about the trials would bring further insight [11], suggesting that even more data sources might be useful in systematic reviews that already include individual participant data.

Given the potential for meta-bias [3], it seems obvious that reviewers should search for and include all relevant and reliable data in systematic reviews. On the other hand, comprehensive searching adds to the time and resources required to complete systematic reviews. Despite the potential value of individual-level data, at this time, it is unclear if the results of systematic reviews using IPD necessarily differ substantively from reviews based on reports of aggregate data. Thus, it is not known if the additional resources required for identifying, obtaining, and analyzing each type of unpublished data are worthwhile. Empirically grounded guidance is needed to guide reviewer choices about the use of data from multiple sources.

### Patient-centered outcomes

Patient-centered outcomes research helps people “communicate and make informed healthcare decisions” (http://www.pcori.org/assets/March-5-Definition-of-PCOR11.pdf), yet many clinical trials and systematic reviews do not fulfill these goals. A primary reason for this deficiency is a historical lack of attention to the selection of patient-centered outcomes for analysis. This project seeks to identify patient-centered outcomes for two systematic reviews using a combination of methods that other reviewers could replicate to improve the patient-centeredness of their research.

Across systematic reviews of a topic, there is a tendency to focus on questions that are answerable and to report outcomes that are available in published reports. This happens even when systematic review authors would prefer to focus on outcomes they consider important but which do not appear in publication. When this kind of availability bias occurs, results published in reports of clinical trials and, thus in systematic reviews, may not be the most meaningful outcomes for people with health problems.

Randomized trials are often expensive (largely because of the effort involved in ensuring high-quality data collection and follow-up), and trialists typically collect more data than they can report in journal publications. If searching for unpublished reports results in the identification of patient-centered outcomes that were recorded but not included in trial publications, then these efforts might improve the quality and utility of systematic reviews by aiding the inclusion of patient-centered outcomes. As far as we are aware, this possibility has never been addressed. Examining such data sources for patient-centered outcomes could reduce the need for additional studies and improve the efficiency of patient-centered outcomes research.

Similarly, reports of clinical trials and systematic reviews typically focus on one time point (e.g., the end of treatment or longest follow-up). From a patient’s perspective, these time points may or may not relate to the natural course or treatment of their problem.

### Objectives

Our objective is to explore the reliability, validity, and utility of incorporating data from multiple data sources. We will assess the impact of using various data sources on effect estimates for efficacy and harms, and on clinical inference, in two high-impact case studies.

To examine the sensitivity of conclusions to the data sources used, we will conduct sequential meta-analyses in which we systematically replace data from less complete sources (e.g., conference abstracts) with data from more complete sources (e.g., journal articles and internal company documents) and with IPD for two systematic reviews. We will evaluate the validity of meta-analyses using these sources by comparing (1) the risk of bias for each analysis and (2) the average effects of meta-analyses based on these sources. We will describe the utility of using additional data sources in meta-analyses, including the information gained by including short reports (e.g., conferences abstracts) and unpublished data (e.g., internal company reports and IPD) in addition to journal articles. We will examine the reliability of sources by comparing outcomes and effects across multiple reports of trials.

## Methods

### Selection of case studies

To evaluate the effect of using data from multiple sources in systematic reviews, we will conduct reviews of the effectiveness and safety of (1) gabapentin (Neurontin®) for neuropathic pain in adults and (2) quetiapine (Seroquel®) for the treatment of depression in adults with bipolar disorder. We will use similar methods for both reviews, as described in this section. The specific inclusion and exclusion criteria will reflect the important clinical issues in each area and are described in the sections that follow. Both reviews will be conducted according to IOM standards [16]. These reviews will be used as case studies to explore the use of multiple data sources.

These cases were selected for several reasons. Firstly, gabapentin and quetiapine are used commonly for these respective conditions, so the included studies will be clinically important. Secondly, pain and depression are associated with several patient-reported outcomes. Self-reported outcomes related to patient perception share common features and complexities in their measurement, so methodological guidance from this project will be relevant to other areas of patient-centered research. Trials in such areas also commonly record outcomes that are not patient-centered [17], and we aim to identify the extent to which the outcomes in multiple data sources are patient-centered. Methodologically, these cases will also allow us to consider different situations that systematic reviewers might face with respect to data from multiple sources.

Gabapentin was initially approved for the treatment of epilepsy. At the time the medication was developed, it was not common to register clinical trials prospectively. Furthermore, much of the use of gabapentin for neuropathic pain has been *off-label* for indications not approved by FDA, and data about the use of gabapentin for these indications were not submitted to regulators to our knowledge. Although we do not expect to find that many trials of gabapentin for neuropathic pain were publicly registered, there is evidence of selective outcomes reporting and publication bias in trials of gabapentin for neuropathic pain [18]. Multiple data sources are available for several trials as a consequence of litigation for which one of the authors (KD) was an expert witness. A list of trials conducted by the developer, internal company documents (*Inferential Analysis Plans*, *Research Reports,* and memos) and databases containing individual patient data were provided by Pfizer to the plaintiffs’ lawyers without codebooks, and these were then given to KD to assist with her testimony.

By comparison, quetiapine was initially approved for the treatment of psychotic disorders and later approved for the treatment of bipolar disorder. Several trials of quetiapine for bipolar disorder were registered prospectively. Although the drug is used off-label, the prescription of quetiapine for bipolar disorder has been largely *on-label* (i.e., this indication was approved by FDA). There is also evidence of publication bias among trials of antipsychotics including quetiapine [19]. These cases thus represent two different and important situations that reviewers might encounter with different types of medications and access to multiple data sources.

### Types of studies

We will include randomized controlled trials. We will exclude N-of-1 trials, observational studies, quasi-randomized controlled trials (e.g., alternating allocation), and non-randomized studies. We will exclude studies in which providers or participants were aware of group assignment (i.e., *open-label* studies). Studies will be considered for inclusion regardless of publication status or language of publication.

Current guidelines suggest that parallel group and crossover studies can be combined for analysis if the crossover design is appropriate for the condition and intervention under investigation [11], though poor reporting often limits their inclusion in meta-analysis [20, 21]. For this review, we will identify crossover studies, but we will not include them in the meta-analysis. Neuropathic pain is relatively stable and likely to return in the absence of effective therapy, but short-term crossover studies may have limited clinical relevance for a chronic condition. Bipolar depression is an unstable condition and antipsychotics are not well tolerated, so withdrawals from the first period of a study could make a second period uninterpretable. Crossover studies that are otherwise eligible will be described in the excluded studies.

We will analyze studies enrolling people who are not taking the study drug prior to the start of the trial (i.e., we will include only studies of people *initiating* treatment with gabapentin or quetiapine). Discontinuation studies will be described but not analyzed. The rationale for this decision is that the efficacy and safety of medications may differ for studies randomizing (1) people who are treatment naïve and (2) people who have responded to a study drug. For example, *discontinuation studies* may enroll people already taking the study drug and randomly assign them to continue taking it or switch to placebo, thus excluding people who do not respond to the treatment and people who experience adverse events and discontinue treatment.

### Comparison interventions

Studies will be included if gabapentin or quetiapine is the only intervention that varies between treatment groups. That is, we will include studies of each medication in combination with other therapies compared with the other therapies alone. In factorial studies making more than one eligible comparison (e.g., A versus B and AC versus BC), we will treat these as separate comparisons (rather than combine intervention and control groups within trials).

Comparisons of different doses or formulations of the same drug, comparisons with other treatments, and discontinuation studies will not be analyzed for this report.

### Identifying patient-centered outcomes

In the funding application for this study, we described plans to select outcomes and time points in collaboration with patient and stakeholder partners. As planned, we created a list of symptoms and outcomes that matter most to patients taking gabapentin for neuropathic pain and to patients taking quetiapine for bipolar depression. We identified patient-centered outcomes to be examined using the following methods:

1. We examined the website “PatientsLikeMe” (http://www.patientslikeme.com/), which is a website to identify and record patient-identified outcomes reported by patients. PatientsLikeMe allows people to enter information about their medical history and interventions they have used. People can describe their experience with interventions, including effectiveness and adverse effects. We did not use the information on effectiveness because it is reported in a format that includes the percentage of people who rated the subjective effectiveness in different categories (e.g., *very effective*, *not effective at all*) without clearly defining outcomes in a way that would allow us to compare this information with the results from the trials. Information about adverse- effects is reported as the six most commonly rated effects in a report for each medication, which we will identify and include in our reviews.
2. We examined the compendium “DRUGDEX” (http://micromedex.com/), which is used by health care providers to make treatment decisions. DRUGDEX is a commercial website. The Centers for Medicare and Medicaid (CMS) may use DRUGDEX ratings to make reimbursement decisions related to medically accepted, FDA unapproved anti-cancer treatment regimens. DRUGDEX contains a list of each drug’s adverse effects by organ system.
3. For pain outcomes, we reviewed the Initiative on Methods, Measurement, and Pain Assessment in Clinical Trials (IMMPACT) consensus recommendations [22]. The mission of IMMPACT is to develop consensus reviews and recommendations for improving the design, execution, and interpretation of clinical trials of treatments for pain (http://www.immpact.org/). The IMMPACT group includes researchers, manufacturers, and people with chronic pain.
4. We used PubMed, patient websites, PCORI-funded projects, and the James Lind Alliance to identify additional outcomes.
5. Patient and stakeholder partners identified outcomes they thought should be included in each review.

We discussed the outcomes identified through all sources and selected those that patient and stakeholder partners thought were most patient-centered. The outcomes for each review are included in the sections that follow.

### Identifying patient-centered time points

Working with patients and clinicians, we also identified time points that are important in these conditions. Neuropathic pain and bipolar disorder are both chronic conditions, and patients with these conditions have indicated that long-term outcomes are more meaningful than short-term outcomes. However, acute treatment is related to long-term management in both cases, so investigators might reasonably focus on either short- or long-term results. For people with bipolar disorder, interventions that are effective for an acute episode may be used prophylactically over longer periods of time. Most studies about bipolar disorder are designed to measure recovery from an episode rather than the long-term prevention of relapses. Because a drug that is ineffective during an acute episode would not typically be continued, long-term studies often randomize people to continue taking a drug or to discontinue a drug to which they responded during an episode of mania or depression. For people with chronic pain, it may be impossible to predict who will respond to a given treatment, so people often try a drug for a short period and continue treatment if it is associated with symptom relief and if it is well tolerated. Indeed, a recent Cochrane review recommends that gabapentin be used this way for the treatment of chronic pain [23]. As above, we discussed possible time points with patient and stakeholder partners. The times they thought were most patient-centered for each review are included in the sections that follow.

### Search methods for identification of studies

We will conduct electronic and additional searches to identify studies, the results of which will be reported following the PRISMA guidelines [24]. We will search for the following types of reports (listed by their approximate level of detail):

1. Study registrations in publicly available databases (e.g., www.clinicaltrials.gov)
2. Study protocols and statistical analysis plans
3. Short reports (e.g., conference abstracts and posters)
4. Summary data posted on trial registries
5. Peer-reviewed journal articles
6. Dissertations (e.g., masters or doctoral theses)
7. Unpublished manuscripts and reports (e.g., reports to funders and clinical study reports)
8. Information sent to regulators (e.g., data sent to FDA)
9. Individual participant data

### Electronic searches

We will search electronic databases, including the Cochrane Central Register of Controlled Trials (CENTRAL), CINAHL, Embase, Lilacs, and PubMed. We will search Medline and PsycInfo for the review of quetiapine for bipolar depression. In addition, we will search the International Clinical Trials Registry Platform Search Portal (ICTRP) and ClinicalTrials.gov to identify study protocols and results [25], using generic drug names and the trade names identified through Micromedex. We will remove duplicates from ClinicalTrials.gov from the ICTRP results.

### Searching other resources

Reference lists of systematic reviews and included studies will be checked for additional reports. We will contact authors of included studies to request additional study reports. We will also contact manufacturers and search their websites to identify reports.

#### Regulatory data

We will search for summary data for studies meeting our eligibility criteria from the FDA website (Drugs@FDA). We will also search the websites of foreign regulators, including the European Medicines Agency (EMA), Medicines and Healthcare Products Regulatory Agency (MHRA, UK), Therapeutic Goods Administration (TGA, Australia), and the Pharmaceuticals and Medical Devices Agency (PMDA, Japan). From each organization’s website, we will download the approval letter and related documents for the relevant indications [26].

On the Drugs@FDA website, we will enter generic drug names as search terms to identify all related products. We will then screen records related to each product to identify potentially relevant documents. Medical and statistical reviews that might include information about the methods of results of eligible trials will be extracted, and we will review the most recent label for information about eligible trials.

We will write to the FDA and EMA to request any information they have about the trials we have identified through our searches (e.g., clinical study reports and individual participant data), and we will request details of any other known studies meeting the inclusion criteria.

#### Individual participant data

For all trials identified, we will request de-identified individual participant data and associated documentation from the study authors and/or sponsor, unless we already have these files through an alternative source. For example, individual participant data for several gabapentin trials have been provided as Microsoft Access files to Kay Dickersin, who provided expert testimony in litigation against Pfizer, but no codebooks were provided with them. We will request further details about these studies (including codebooks to verify definitions of variables) as appropriate given ongoing litigation and settlement agreements.

We will search for data that have been made publicly available by manufacturers (e.g., through their websites). We will also search for data that have become available through other means (e.g., litigation) using the Drug Industry Document Archive (DIDA), Yale University Open Data Access (YODA), and PsychRights.org.

### Selection of studies

Two reviewers will independently screen titles and abstracts identified by the electronic searches to determine which are eligible for inclusion in the review. We will then obtain and independently screen the full text of all potentially relevant studies to determine whether they meet the inclusion criteria. If investigators disagree about the eligibility of a report, they will discuss the disagreement with a third investigator to reach consensus about the study’s eligibility.

If a study cannot be included or excluded based on the information available in all reports associated with the study, we will contact the study authors and/or sponsor for more information to determine eligibility for our review.

During the study selection process, we will not be masked to study authors, institutions, journal of publication, or results.

Results of the search will be documented using modified PRISMA flowcharts [24].

### Outcomes

Reports may include both systematically recorded outcomes (i.e., those that have been recorded using standardized measures given to all participants) and spontaneously recorded outcomes (e.g., unexpected outcomes reported by participants or providers).

### Systematically recorded outcomes

From each report, we will record the outcomes, and we will record if they are identified as “primary,” “secondary,” and “safety,” using another definition, or not defined classified.

We will record five elements for each outcome as they are described in each report [27, 28]:

1. Domain (outcome title);
2. Specific measure (specific scale or instrument);
3. Specific metric (format of the outcome data, such as value-at-a-time or mean change from baseline);
4. Method of aggregation (how data from each group will be summarized, such as mean or percent); and
5. Time point (e.g., weeks or months since randomization).

In addition to these elements, we will record details about methods of analysis (e.g., handling of missing data) and the definition of the population for analysis (e.g., study completers or people starting treatment).

We will extract and analyze results for key domains. These domains will be selected because they are (1) commonly measured, (2) likely to be reported selectively based on previous research, or (3) important to patients. The reporting of other outcomes will be described, but meta-analyses will not be conducted.

### Spontaneously reported adverse events

In choosing a treatment for neuropathic pain or bipolar disorder, differences among available drugs in risk of adverse events may be more important than differences in average efficacy.

Adverse events can be recorded systematically using tests or questionnaires; however, trials often record only adverse events that patients report to doctors or investigators (e.g., on a case report form), which produces data that are difficult to analyze within and across trials. A problem related to this method of data collection is that adverse events may be reported in ad hoc and selective fashion in clinical trials; thus, systematic reviews may not pre-specify adverse outcomes or collect adverse event data systematically. Even when systematic review authors make every effort to assess effectiveness outcomes using scientific methods, they may not be able to apply the same standards to the synthesis of adverse events [29].

In these reviews, we will extract detailed information about adverse events from all reports to identify similarities and differences across reports of clinical trials. In addition to other relevant information (such as the number of people assigned and included in each analysis), we will extract the following information about adverse events:

1. Number (proportion) of participants experiencing one or more adverse events
2. Number (proportion) of participants who discontinued treatment because of adverse events
3. Number (proportion) of participants who discontinued treatment for any reason
4. Number of serious adverse events (i.e., those that could be classified as serious by the FDA, including death, life-threatening events, hospitalization, disability or permanent damage, and important medical events)
5. Specific adverse events. Where possible, these will be recorded following the classification systems used by developers at the time trials were conducted. Most clinical trials would have been analyzed using either the Coding Symbols for Thesaurus of Adverse Reaction Terms (COSTART) or the Medical Dictionary for Regulatory Activities (MedDRA), depending on the time they were conducted.
  1. For COSTART version IV [30], these are the following:
    - Body system: “Essentially anatomic, this body system classification is sometimes the basis of search strategy. The classification is hierarchical in nature.”
    - Body system subcategories
    - Mid-level system: “a mid-level pathophysiologic classification of COSTART for purposes of categorizing and retrieving information based on disease associations.” “This section is hierarchical in arrangement, allowing one to be very general or more specific and is a convenient strategy for searching for drug-induced disease.”
    - Mid-level system subcategories
    - Preferred term: A 20-character code used to identify events
  2. For MedDRA [31], these are the following:
    - System organ class (SOC): “the highest level of the hierarchy that provides the broadest concept for data retrieval.” Data are grouped by etiology, manifestation site, and purpose. To avoid double-counting preferred terms (PTs) assigned to more than one SOC, we will use only the “primary” SOC.
    - High-level group term (HLGT): “a superordinate descriptor for one or more HLTs related by anatomy, pathology, physiology, etiology, or function”
    - High-level term (HLT): a superordinate category that “links PTs related to it by anatomy, pathology, physiology, etiology, or function.”
    - Preferred term (PT): “a distinct descriptor (single medical concept) for a symptom, sign, disease, diagnosis, therapeutic indication, investigation, surgical, or medical procedure, and medical, social, or family history characteristic.”
    - Lowest level term (LLT): synonyms for a preferred term.

Results coded using COSTART will be analyzed at the level “Preferred term,” “Mid-level system,” and “Body system.” Results coded using MedDRA will be analyzed at the level “Preferred term” and above. Additionally, we will use searches designed to identify clusters of related symptoms that may not be identifiable using the standard hierarchy [32].

Adverse events could have been recorded using another hierarchical system, such as WHOART, and results will be analyzed using these systems where appropriate. For reports that do not describe adverse events using a structured classification system, we will record events as they are described in the reports. Where possible, we will also compare the terms given to the original reporter (e.g., as written on a case report form) and the preferred terms with which these were associated.

### Time points for analysis

We will extract the time at which each outcome was assessed as described in each report, and we will describe the planned duration of the included trials.

Effectiveness outcomes (i.e., data about benefits) will be organized into results at 8 weeks post-randomization (time window 4–13 weeks), 18 weeks (time window 14–22 weeks), 27 weeks (time window 23–31 weeks), and longer times where possible. For each review, we plan to meta-analyze data for the 8-week time window because patients may decide if they wish to continue using a medication during this interval. Additionally, we expect to have the most data for analysis in the short-term time window. If sufficient data are available, we will also analyze outcomes at other times. For each time window, we will describe how all elements for these outcomes were reported in each source.

For each of these time windows, key outcomes will be analyzed in sequential meta-analyses comparing combined effects using different data sources. If results are reported for a study at multiple times within a window, we will use the time closest to 8, 18, or 27 weeks for meta-analysis.

Adverse events will be analyzed for the same times as the effectiveness data where possible. Additionally, we will analyze adverse events occurring closer to randomization (e.g., 3–4 weeks), which may be important for understanding early discontinuation and compliance.

Because we will analyze only comparators that do not include the test intervention (e.g., placebo), we do not expect to find many long-term studies for both ethical and practical reasons. For example, people who do not respond to an intervention or placebo after several months may be unlikely to continue participating in a trial; even if a trial were to continue beyond acute treatment, missing data would make long-term results difficult to interpret.

### Data collection

#### For aggregate data

Data collection forms will be developed and entered in the Systematic Review Data Repository (SRDR). These will be made available through SRDR at the end of the study.

Data collection forms will be pilot tested. After the initial versions of the forms have been finalized, data will be extracted from each report into SRDR. For studies associated with multiple reports (e.g., journal article and internal company documents), data will be extracted from each report for comparison. From each source, we will record details about the report, study design, start and end dates, inclusion and exclusion criteria, characteristics of each intervention, participant demographic and clinical characteristics, outcomes, risk of bias, and sponsor.

Data will be extracted independently by two reviewers. Discrepancies will be resolved by consensus and through discussion with a third reviewer if necessary. The final dataset will be made publicly available through SRDR at the end of the study.

#### For individual participant data

Individual participant data will be accepted in any format, and these will be analyzed if possible. Where it may be possible to obtain data in various formats, we will state our preference for receiving data based on our familiarity with relevant software and the desirability for concordance across studies.

Because we do not have codebooks for some gabapentin databases, it is not clear that we will be able to include all IPD from available sources in our analysis. Where possible, we will compare the available databases with case report forms (which often show how and when data were recorded) and statistical analysis plans (which often show how data were coded and analyzed) to identify the variables contained in the databases. From our initial review, we can see that the databases include participant numbers, diagnostic information, information about study site, dates of medical visits, and details about the outcomes and the types and times of adverse events.

We have also identified some individual participant data in clinical study reports (CSRs) about quetiapine for bipolar depression. These reports mainly include aggregate results, but some reports also contain data for individual patients, which are organized in tables by participant number. We are currently working to extract information from these reports in an analyzable format. As with gabapentin, the reports include diagnostic information, information about study site, dates of medical visits, and details about the types and times of adverse events. Where possible, we will use ABBYY FineReader software to extract data from PDF files into spreadsheets for analysis [33].

Whether codebooks are available through the authors or we have had to reconstruct them, we will check the data for accuracy. We will first attempt to recreate tables found in the available reports to examine whether we have correctly identified study variables, including those associated with baseline characteristics and results. Where data have been reformatted for analysis, will also check a sample of the cells against the original source for accuracy.

Trials of these drugs have been conducted over two decades, and it may not be possible to include all IPD in our analyses from individual investigators because of the number of datasets received or because some data formats cannot be merged for analysis (e.g., it may not be possible to combine older formats and newer formats). If we are not able to include all of the data in the meta-analysis because they cannot be synthesized in the time available, we will describe datasets not included in the analysis.

### Confidentiality

De-identified data will be collated in a common database for each review using fields that are consistent across trials where possible. Until completion of our study, data will be kept on a local network to which only people working on this project have access. Unless we find that participants could be identified using these data, we will make databases and codebooks publicly available following the completion of our study.

In the event that any personally identifiable information is found, identifying information will be removed from the data following Health Insurance Portability and Accountability Act (HIPAA) guidelines before sharing the files with others.

We obtained IPD for trials of gabapentin as a result of litigation for which Kay Dickersin was an expert witness. Databases (Microsoft Access files) and documents (PDF files) containing IPD were provided by Pfizer to the plaintiffs’ lawyers without codebooks, and these were then given to Kay Dickersin to assist with her testimony. The data have been unsealed, and Pfizer has waived claims of confidentiality. The quetiapine clinical study reports include dates of medical tests and narratives about adverse events that describe the age, sex, and race of participants; we have also located patient initials in some of the reports. The clinical study reports that include individual participant data for quetiapine were made publicly available by the plaintiffs’ attorneys following a product liability lawsuit, and these are already available on the Internet.

### Assessment of risk of bias in included studies

To compare the completeness of reports with respect to the methodological information they contain, each report will be assessed using the Cochrane Collaboration Risk of Bias Tool [34]. Two reviewers will independently rate each report for risk of bias related to sequence generation, allocation concealment, masking of participants, masking of outcome assessors, masking of providers, and incomplete data. We will not rate risk of selective outcomes reporting in each report, but we will rate each trial for risk of reporting bias. For each domain, risk of reporting bias will be described as high, low, or unclear. Discrepancies will be resolved by consensus and through discussion with a third reviewer if necessary.

### Dealing with missing data

Missing data are unrecorded values that could be meaningful for the analysis and interpretation of a study [35]. In clinical trials, data may be missing for outcomes or for covariates. For outcomes, a participant who skips questions on a measure at a given time point may complete related questions and outcome measures; in such cases, *missing items* (i.e., questions) may be imputed to calculate the overall scores for measures (i.e., questionnaires). In other cases, outcome measures may be missing in their entirety; *missing outcome measures* may be related to missed assessments (e.g., missed visits) or to discontinuation (e.g., study dropout). For all outcomes, we will report the amount of missing data and the reasons for missinginess. Additionally, the following sections describe how we will handle missing outcomes in reports of aggregate data and how we will handle IPD with missing outcomes, including *missing items* and *missing outcome measures*.

#### In reports of aggregate data

When aggregate analyses of continuous outcomes are reported only for people providing outcome data as well as controlling for missing data (e.g., using multiple imputation), we will analyze the latter. For dichotomous measures of treatment efficacy, we will conduct an analysis in which we assume that participants did not respond to treatment if their outcomes are missing. For dichotomous measures of adverse events, we will conduct an analysis in which we assume that participants who took at least one dose of the assigned medication were at risk of those events. For dichotomous outcomes, we will conduct sensitivity analyses to evaluate how the results are affected by different assumptions about missing data.

For secondary (sensitivity) analysis, we will use the pattern-mixture approach accounting for the uncertainty due to missing data [36–39]. We will calculate the *adjusted* treatment effects for each outcome for which results are reported without imputation, then synthesize adjusted and unadjusted treatment effects via standard meta-analysis across all studies. Adjusted treatment effects are related to the informative missingness defined as a ratio (or difference) between missing and observed treatment effects. We will implement this approach under various scenarios by considering a wide range of the informative missingness (that is, we will assume the missing and observed treatment effects are similar or different).

#### For individual participant data

For all trials for which we have IPD, we will attempt to replicate the analyses performed by the study authors. Since the time that most studies of gabapentin for neuropathic pain and quetiapine for bipolar depression were conducted, researchers have developed new techniques to deal with missing outcome measures. We will use current best practices for handling missing data to determine if reanalysis of individual participant data following current best practice might lead to conclusions that differ from those of the original analyses.

#### Missing items in individual participant data

For outcome measures (i.e., questionnaires) with multiple items (i.e., questions), we will attempt to determine how sensitive the results might be to missing items. For each treatment group in each trial, we will describe the mean, maximum, and minimum number of missing items for each outcome measure where possible. We will impute missing items using methods described by the authors where possible. Additionally, we will impute missing items for standardized scales using standard coding techniques in the event that the authors did not impute missing items using standard methods.

#### Missing outcome measures in individual participant data

We anticipate that two analyses will allow us to replicate the handling of missing outcome measures (e.g., missing visits) for most of the analyses conducted by the authors, *complete case analysis* and *last observation carried forward*. In the presence of missing data, comparing imputed results (i.e., using best current methods, as described below) with a complete case analysis is important for evaluating the consequences of missing data and imputation.

1. Complete case analysis: for each outcome measure, we will include participants who completed a specific outcome measure at a specific time point of interest (e.g., when looking at the Short Form-36 (SF-36) at 6 weeks, we will include all individuals who completed the SF-36 at that time point). In addition, we will exclude participants who did not complete enough items to derive a summary score for the outcome measure at the specific time point.
2. Last observation carried forward (LOCF): for each outcome, we will conduct an analysis that includes all participants who completed that outcome measure at baseline assessment. If a participant did not complete an outcome measure at a given point in time, or if a participant did not complete enough of an outcome measure to derive a summary score for that measure, we will impute the outcome measure by carrying forward the last observation for that measure. Although single imputation is no longer recommended for handling missing data, this analysis will allow us to compare our results with the results calculated by the trialists [40, 41].

#### Best current methods

For the meta-analysis, we will estimate the treatment effects using multiple imputations to impute missing outcome measures and to account appropriately for the uncertainty because of missing data [40, 42]. We will conduct imputations separately for each of the trials. For each trial, we will impute both individual-level covariates measured at baseline (including participant characteristics and baseline measures of outcomes) and outcomes measured at every follow-up time point together using a “multiple imputation by chained equations” (MICE) approach, as implemented in the *mi impute chained* command in Stata [43]. MICE cycles through the variables, imputing each variable one at a time, by fitting a model of that variable as a function of the other variables in the imputation procedure. This process of imputing each variable one at a time is then repeated until the algorithm reaches convergence. The MICE approach allows each variable to be modeled according to its own distribution, and it can easily handle data complications such as variables with restricted ranges (e.g., age will be at least 18 years and cannot exceed 120 years). In general, we will use logistic regression for binary variables, multinomial logistic regression for categorical variables, Poisson regression for count variables, and a linear regression for continuous variables. For continuous variables that are not normally distributed, we will transform the data to be normally distributed to improve the fit of the imputation models, which will be linear regression for continuous variables. If we cannot transform a continuous variable to be normally distributed, we will use predictive mean matching [44].

The specific variables included in the imputation for each trial will include treatment group, demographic characteristics (e.g., sex and age), outcome measures at baseline and each recorded follow-up (e.g., daily pain score, present pain intensity, SF-36, SF-MPQ), as well as other clinical measures that are available across studies (e.g., heart rate, blood pressure). We will not include race in the model. In the case of convergence problems with MICE resulting from the number of variables or collinearity, we will use stepwise selection models to assist with the imputations. If there is no computational limit, we will create 100 imputations for each trial to achieve high efficiency [45]. We will estimate the treatment effect within each imputed dataset separately, and then we will combine the estimates for each trial using the standard multiple imputation combining rules [42]. Then, we will synthesize the combined treatment effect estimates across all studies as described below (“Data synthesis“ section).

Imputing all measures together as we will do (i.e., both baseline and outcome variables) utilizes all data available for each participant. For example, for a participant with just one missing follow-up time point, we will impute that missing value using information from observed values for that participant at other time points. In addition, it is seen as the best practice for imputation in clinical trials to include outcome measures with all the covariates in the imputation process, as we are planning to do here (and as described above) [46, 47].

Although White et al. recommend restricting analysis to individuals reporting outcome measurement after imputing covariates and outcomes together, we will estimate treatment effects using all individuals, including those with imputed outcomes [48]. In our IPD meta-analysis, restricting to individuals with observed outcomes would result in the same treatment effect estimates as obtained under the complete case analysis.

#### Sensitivity analyses

1. Pooled imputation: we will conduct a sensitivity analysis, modified from our MICE approach. In this sensitivity analysis, we will use a pooled imputation model in which we implement the multiple imputation procedure across all trials simultaneously. This imputation model will include outcome measures and other variables available for individual participants in at least three (50 %) of the studies for which we have IPD for gabapentin or in both of the studies for which we have IPD for quetiapine. For each review, the imputation will be carried out using a merged dataset that combines multiple studies. This imputation procedure will also include study indicators in the imputation models to account for heterogeneity across studies. One limitation of this approach is that it will not be helpful if reported covariates substantially differ across trials
2. Imputation with non-ignorable missingness: multiple imputation, as implemented following the procedures described above (“Best current methods“ section), assumes that missingness depends only on the observed data (i.e., missing at random) and that the missingness is not related to variables that were not measured. As a secondary sensitivity analysis, we will consider non-ignorable missingness (missing not at random (MNAR)), which assumes that missingness depends on both observed and unobserved data. To do this, we will model the outcomes and missingness pattern jointly using selection or pattern-mixture model techniques [42]. Using this model, we will estimate the treatment effect in each study, and we will conduct a meta-analysis to combine the estimated treatment effects across all studies as described below (“Data synthesis“ section). Furthermore, we will conduc simulation studies to investigate the impact of different missingness mechanisms with various missingness rates on treatment effect estimation in meta-analyses.

### Assessment of heterogeneity

To assess clinical and methodological heterogeneity, we will present the characteristics of studies in tables and describe the similarity of participants, interventions, outcomes, and methods across studies.

To assess statistical heterogeneity, we will:

1. Visually inspect forest plots to see if the confidence intervals of individual studies have poor overlap—a rough indication of statistical heterogeneity;
2. Calculate the *I*^2^ statistic, which describes the percentage of observed heterogeneity that would not be expected by chance [49]. By convention, we will describe an analysis as having substantial statistical heterogeneity if its *I*^2^ statistic is greater than 50 %; and
3. Calculate tau, which captures the amount of heterogeneity on the same scale and unit of the outcome measure.

### Data synthesis

For dichotomous outcomes (other than spontaneously reported adverse events), we will calculate risk ratios (RR) within studies and the summary risk ratio across studies [34]. For continuous outcomes measured on the same scale in all trials, we will calculate the weighted mean difference (WMD). For continuous outcomes measured on more than one scale, we will calculate the standardized mean difference (Hedges *g*). In studies reporting more than one measure of a domain, the most common measure across studies will be selected for analysis to minimize methodological heterogeneity. If some studies do not include the most common measure, we will select the most similar measure (rather than average treatment effects within studies).

In meta-analyses that include both aggregate and individual participant data, we will conduct two-stage meta-analyses [50, 51]. Specifically, individual participant data will be analyzed for each trial and then the results will be combined with aggregate data across all studies, assuming that aggregate and individual participant data estimate the same treatment effects [14, 52]. This enables us to borrow information from both levels of data and is the best use of all existing data.

All meta-analyses will be calculated using random-effects and reported with 95 % confidence intervals (CI) [53–55].

Spontaneously reported adverse events will be described in tables, but these will not be analyzed statistically. We will record the number of events reported in the test (i.e., gabapentin or quetiapine) and comparator (e.g., placebo) groups of every trial report, and we will report the total number of events for all test and comparator groups for each data source. Where possible, we will sum individual participant data at the “Preferred term” level and above using the COSTART and MedDRA classification systems (see “Spontaneously reported adverse events“), and we will record data from other sources as they were reported.

Study design, participant characteristics, and treatment characteristics may affect results, so we will conduct a priori subgroup analyses to examine moderators using aggregate data or individual participant data where possible [56]. We will investigate differences between subgroups using a test for interaction (*p* < 0.1 considered relevant). Residual heterogeneity will be quantified using *I*^2^, which we will describe as substantial if *I*^2^ is greater than 50 %.

### Comparing multiple sources

For pre-specified outcomes, we will produce a table showing the results from each study, which will be combined in an overall analysis including results from each data source, including the following:

1. Short reports (e.g., conference abstracts and posters);
2. Peer-reviewed journal articles (about one or more trials);
3. Summary data posted on trial registries;
4. Dissertations (e.g., masters or doctoral theses);
5. Unpublished manuscripts and reports (e.g., reports to funders, clinical study reports);
6. Information sent to regulators (e.g., data sent to FDA); and
7. Individual participant data.

We will examine if the results show evidence of reporting bias by comparing multiple data sources for studies associated with more than one report. For studies with both sources of data, we will compare the following:

1. Results from individual participant data compared with CSRs; and
2. Results from CSRs compared with published results.

We will then conduct a series of meta-analyses to explore the impact of multiple data sources on the overall results. We will analyze these results by sequentially adding or replacing data as follows:

1. Including only results from short reports (e.g., conference abstracts and posters);
2. Replacing data from short reports (step 1) with data from publications in peer-reviewed journals and adding data from studies reported in peer-reviewed publications but not short reports;
3. Replacing data from publications (step 2) using summary data obtained from the authors or manufacturers, regulators, or trial registries for the trials included above;
4. Adding data (to step 3) from unpublished trials using data obtained from regulators, or trial registries;
5. Adding or replacing data (from step 4) for unpublished trials with aggregate data obtained from the authors or manufacturers (e.g., clinical study reports);
6. Replacing the best available aggregate data for all studies (step 5) with individual participant data where available.

If more than one report of a particular type is available for a trial (e.g., several peer-reviewed publications), we will include data from all of them in the meta-analyses. We will note discrepancies where they exist, and we will analyze results from the main report of a trial if the main report and other reports are discrepant. If reports include data for more than one trial, we will include data reported separately for each trial; we will not include combined results for these analyses unless no other estimates are available for the included studies.

To compare studies that have only been reported in short reports (e.g., a conference abstract or poster) with studies that have been reported in greater detail, we will conduct one further step in this sequence:

7. Removing short reports (e.g., conference abstracts and posters).

We will investigate if the results for published and unpublished studies differ using the best available data for each.

By analyzing results in this sequence, we hope to identify and to quantify differences that are attributable to (1) information about additional outcomes examined (*selective outcome reporting*), (2) information from more than one source for a data item (*competing information*), and (3) information about previously hidden trials (*publication bias)*.

For the main outcome in each review, we will explore the distribution of possible effects by calculating all combinations of reports for each outcome and reporting the range of observed means. We will explore the extent to which these estimates are influenced by the inclusion or exclusion of particular reports, paying particular attention to deviations from the mean that represent clinically important differences in the outcomes under investigation.

### Criteria for selecting studies of gebapentin for neuropathic pain in adults (CRD42015014037)

#### Background

Peripheral neuropathy occurs when there is damage to the peripheral nervous system, the array of nerves that transmit information from the central nervous system (brain and spinal cord) to other parts of the body. There are hundreds of types of peripheral neuropathy, and the ways that individuals are affected (impaired function and symptoms) depends on the type of damage. Painful, chronic neuropathies can be caused by trauma, systemic diseases (e.g., diabetes, kidney disorders, cancer, hormone imbalances, and vitamin deficiencies), infections, immune disorders, chemotherapy, and other conditions. Between 3 and 10 % of the population may be living with painful neuropathy, and painful neuropathy affects 30 to 50 % of people with diabetes in particular [57]. Painful neuropathy and its sequelae, such as loss of sleep, result in reduced quality of life and high health care costs. For example, the cost of diabetic peripheral neuropathy has been estimated to be US$5 to US$14 billion, which accounts for up to 27 % of the direct medical costs associated with diabetes [58].

Gabapentin (Neurontin®) was approved by the FDA for the treatment of epilepsy in 1993, and it was approved in 2002 for the treatment of post-herpetic neuralgia (i.e., residual pain in people who have had shingles). It is used “off-label” for a variety of symptoms, including neuropathic and other types of pain.

#### Types of participants

Studies with adults (18 years and older) with neuropathic pain will be included without restriction by setting (e.g., hospital or outpatient) or comorbidity, except that participants requiring ventilator support will be excluded because effects may not be comparable to ambulatory populations. Studies including people with neuropathic pain as well as other conditions will be included if disaggregated data are available (in a report or from the authors) such that outcomes can be extracted separately for people with neuropathic pain (i.e., either individual participant data or aggregate data).

We will include participants considered to have neuropathic pain secondary to one or more of the following underlying conditions:

- Cancer (malignancy or chemotherapy induced);
- Central stroke;
- Complex regional pain syndrome;
- Diabetes mellitus;
- Guillain-Barré syndrome;
- Herpes zoster infection;
- HIV infection;
- Multiple sclerosis;
- Nerve compression or entrapment including carpal tunnel syndrome and vertebral disc prolapse;
- Phantom limb pain;
- Radicular pain, including radiculopathy associated with spinal stenosis;
- Spinal cord injury;
- Trauma; and
- Trigeminal neuralgia

We will exclude participants considered to have pain resulting from the following conditions:

- Chronic low back pain other than radicular pain;
- Chronic pelvic pain (which is multifactorial in etiology and not solely of neuropathic origin);
- Fibromyalgia (there is no consensus on whether this is neuropathic pain);
- Lyme borreliosis;
- Migraine;
- Osteoarthritis (pain is considered to be nociceptive in nature);
- Pre- or post-operative acute pain (e.g., following thoracotomy or spinal fusion) or pain following vaginal delivery;
- Restless leg syndrome;
- Spinal stenosis without radiculopathy.

#### Types of interventions

We will include trials of oral gabapentin alone, or oral gabapentin in combination with another medication, with a daily dose of 300 mg gabapentin or above. Studies in which the dose of gabapentin was escalated until pain relief was achieved will be eligible. Studies of gabapentin enacarbil, a prodrug, will be excluded.

#### Outcomes for sequential meta-analysis

Following consultation with patients and clinicians, a review of existing trials and recommendations from the Initiative on Methods, Measurement, and Pain Assessment in Clinical Trials (IMMPACT) group [22], the following key outcomes were selected and will be extracted from each report and analyzed in a sequential meta-analysis to compare combined effects for meta-analyses using different data sources. Other outcomes will be extracted but not meta-analyzed as described below.

1. Pain intensity: severity of daily pain as measured by any pain scale or instrument. This is often assessed using a scale with values from 0 (“no pain”) to 10 (“worst possible pain”), completed daily. Depending on how pain was measured and reported in the source documents, we will analyze it as a continuous outcome or a categorical outcome.
  - Improvement in pain
    i. 50 % improvement: proportion of participants in each group with ≥50 % reduction in mean daily pain intensity for a period of time (e.g., 1 week) prior to treatment compared with most recent period of time or a functionally similar definition.
    ii. 30 % improvement: proportion of participants in each group with ≥30 % reduction in mean daily pain intensity for a period of time (e.g., 1 week) prior to treatment compared with most recent period of time or a functionally similar definition.
  - Change in pain intensity: mean change in daily rating for a period of time (e.g., 1 week) prior to treatment compared with mean daily rating for most recent week or a functionally similar definition.
  - Patient global impression of change: proportion of participants in each group reporting “very much improved“ or “much improved”
  - Clinician global impression of change (CGIC or CGI): proportion of participants “very much improved” or “much improved”
2. Pain interference: the extent to which pain prevents normal functioning as measured by any pain scale or instrument (e.g., the Multidimensional Pain Inventory Interference Scale). We will analyze pain interference as a continuous outcome such as mean change in interference for a period of time (e.g., 1 week) prior to treatment compared with mean daily rating for the most recent period of time or a functionally similar definition.
3. Sleep disturbance: difficulty sleeping as measured by any scale or instrument. This is often assessed using a numeric scale, completed daily, describing how pain interfered with sleep during the last 24 h; scores may range from 0 (“does not interfere with sleep”) to 10 (“completely interferes with sleep”). We will analyze sleep disturbance as a continuous outcome such as mean change in daily rating for a period of time (e.g., 1 week) prior to treatment compared with mean daily rating for the most recent period of time or a functionally similar definition.
4. Quality of life (QOL): health-related quality of life as measured by any scale or instrument will be analyzed as a continuous outcome (e.g., change in QOL assessed using the mean difference from baseline measured on the Short Form-36). For measures with multiple subscales, we will include the total in the main analysis if possible.

In addition to those outcomes included in a sequential meta-analysis, spontaneously reported adverse events will be recorded and aggregated using the methods described above.

#### Outcomes for descriptive analysis

Definitions of the following outcomes, including the elements described above [27, 28], will be extracted from each report. We will use these data to evaluate the completeness of reporting and to identify differences in outcomes among reports.

1. Mood, including but not limited to:
  - Measures of depression (e.g., Beck Depression Inventory)
  - Measures of anxiety (e.g., State-Trait Anxiety Inventory)
  - Measures of overall mood state (e.g., profile of mood states) or psychiatric functioning (e.g., CORE-OM)
2. Lab tests (e.g., hemoglobin or glucose levels)
3. Evoked pain (e.g., allodynia or hyperalgesia)
4. Consumption of concurrent medication for pain (sometimes described as “escape” or “rescue” medication)
5. Time-to-event data related to any domains above

#### Baseline data to record

To describe the characteristics of participants in each study, we will extract demographic and clinical characteristics from each report. For the total sample, and for each group where possible, we will record the following:

1. Age
2. Sex
3. Weight
4. Self-reported race/ethnicity (percentage of non-white)
5. Drug and alcohol use
6. Study location (country and state or county)
7. Previous response or non-response to medication (e.g., gabapentin, other pain medications)
8. Pain condition
9. Duration of pain (i.e., time since onset of the condition)
10. History of anxiety or depressive disorder

### Subgroup analysis and investigation of heterogeneity

We will summarize the characteristics of included studies and describe potential sources of clinical and methodological heterogeneity among them, and their potential influence on results.

We will also conduct the following subgroup analyses:

1. Sex (men versus women)
2. Pain condition (post-herpetic pain; diabetic neuropathy; other types of pain)
3. Daily dose (variable dose studies; fixed doses of 300–900, 901–1800, 1801–2700, and 2701 mg or more)

### Searching for published literature

Databases and trial registries will be searched using the terms in Appendix 1.

### Criteria for selecting studies of quetiapine for bipolar depression in adults (CRD42015014038)

#### Background

Bipolar disorder has a lifetime prevalence of 1–4 % [59, 60]. It is characterized by episodes of depression and at least one episode of mania or hypomania [61]. Work, family life, and social life are impaired significantly by depressive episodes [62, 63]. Because of their severity and frequency, depressive episodes account for three times more time spent with disability than manic episodes [61, 64, 65]. People with bipolar disorder are at increased risk of suicide compared with the general population and compared with people who have other mental health problems [66, 67].

Antipsychotics were developed for the treatment of acute psychotic episodes, including bipolar mania, for which there is evidence of short-term efficacy [68]. Quetiapine (Seroquel®) is an antipsychotic that is derived from dibenzothiazepine. It acts as an antagonist at serotonergic 5-HT_2_ receptors and dopaminergic D2 receptors in the central nervous system, but the method by which it might function as an antidepressant remains unclear [69, 70]. It is currently recommended as a first-line choice for treatment of acute bipolar depression by existing guidelines [71, 72], although it is associated with outcomes that are undesirable to patients including daytime sleepiness, cognitive impairment, loss of libido, and rapid weight gain. Quetiapine and other antipsychotics are also associated with serious adverse events, including cardiac and metabolic effects and extrapyramidal symptoms [73, 74].

#### Types of participants

Studies that include adults (18 years and older) with a current episode of depression will be included without restriction by setting (e.g., hospital or outpatient). Participants must have been diagnosed with bipolar disorder (type I or II) using DSM-III, DSM-IV, DSM-V, ICD-9, or ICD-10 criteria or an equivalent structured diagnostic interview. Studies that included only participants described as having the “rapid cycling” subtype will be excluded.

Studies including participants with other disorders (e.g., major depressive disorder and other serious mental illnesses such as schizophrenia spectrum disorders or substance abuse) will be included if disaggregated data are available (in a report or from the authors) such that outcomes can be extracted separately for people with bipolar disorder (i.e., either individual participant data or aggregate data).

Studies including only participants with both bipolar disorder and comorbid disorders (e.g., anxiety or substance misuse) will be included.

#### Types of interventions

We will include studies comparing oral quetiapine (including extended release) with a daily dose of 100 mg or above. Studies of norquetiapine, a metabolite of quetiapine, will be excluded.

#### Outcomes for sequential meta-analysis

Following consultation with patients and clinicians and a review of existing trials, the following key outcomes were selected and will be extracted from each report and analyzed in a sequential meta-analysis to compare combined effects for meta-analyses using different data sources. Other outcomes will be extracted but not meta-analyzed as described below.

1. Depression: symptoms of depression as measured by any scale or instrument. Depending on how depression was measured and reported in the source documents, we will analyze it as a continuous outcome or a categorical outcome.
  - Improvement in symptoms (e.g., proportion of participants reporting ≥50 % reduction in depression as measured using the Hamilton Rating Scale for Depression (HAM-D) or Montgomery-Åsberg Depression Rating Scale (MADRS))
  - Change in symptoms of depression (e.g., mean difference from baseline on the HAM-D, MADRS, or another depression rating scale)
2. Functioning: ability to participate in social, occupational, and family life as measured by any scale or instrument will be analyzed as a continuous outcome (e.g., change from baseline on the Global Assessment of Functioning scale).
3. Quality of life: health-related quality of life as measured by any scale or instrument will be analyzed as a continuous outcome (e.g., change in QOL assessed using the mean difference from baseline measured on the Short Form-36). For measures with multiple subscales, we will include the total in the main analysis if possible.
4. Anxiety: symptoms of anxiety as measured by any scale or instrument. Depending on how anxiety was measured and reported in the source documents, we will analyze it as a continuous outcome or a categorical outcome.
  - Improvement in symptoms (e.g., proportion of participants reporting ≥50 % reduction in anxiety as measured using the Hamilton Rating Scale for Anxiety)
  - Change in symptoms of anxiety (e.g., mean difference from baseline on the HAM-A or another anxiety rating scale)
5. Sleep
  - Proportion of participants using sleep medication
  - Change in sleep (e.g., mean difference from baseline on the Pittsburgh Sleep Quality Index, HAM-D insomnia items, or another measure of sleep)
6. Weight gain (we will combine measures of weight and body mass index because the height of adults is not expected to change during clinical trials.)
  - Measured on a continuous scale, e.g., mean change from baseline or a value-at-a-time
  - Measured categorically such as proportion of participants gaining 2 or 5 % of their baseline weight
7. Psychiatric hospitalization (e.g., proportion of participants admitted to hospital)
8. Suicide
  - Proportion of participants completing suicide
  - Proportion of participants attempting suicide
  - Proportion of participants with suicidal intent or suicidal ideation

In addition to those outcomes included in a sequential meta-analysis, spontaneously reported adverse events will be recorded and aggregated using the methods described above.

#### Outcomes for descriptive analysis

This review focuses on the use of quetiapine for acute episodes, so adverse events associated with long-term use might not be observed. We do not expect to find evidence about outcomes like cataracts that develop over a period longer than the duration of an acute depressive episode. In acute treatment trials, blood tests and vital signs may be monitored to identify increased risk of adverse events, including serious adverse events.

Definitions of the following outcomes, including the elements described above [27, 28], will be extracted from each report. We will use these data to evaluate the completeness of reporting and to identify differences in outcomes among reports.

1. Metabolic effects
  - Proportion of participants with new diagnosis of diabetes mellitus type II
  - Waist circumference
    i. Measured on a continuous scale, e.g., mean change from baseline or a value-at-a-time
    ii. Measured categorically such as proportion of participants with increase ≥5 cm
  - Fasting glucose
    i. Measured on a continuous scale, e.g., mean change from baseline or a value-at-a-time
    ii. Measured categorically, e.g., proportion of participants with fasting glucose ≥100 mg/dL (hyperglycemia) and proportion of participants with fasting glucose ≥126 mg/dL
2. Endocrine effects
  - Proportion of participants reporting loss of interest in sex or sexual dysfunction (e.g., impotence, anorgasmia)
  - Proportion of women reporting irregular or missed menstrual cycle
  - Proportion of men reporting gynecomastia (swelling of the breast tissue)
  - Serum prolactin levels measured on a continuous scale, e.g., mean change from baseline or a value-at-a-time
3. Extrapyramidal symptoms (tardive dyskinesia, dystonia, akathisia)
  - Incident cases: proportion of participants experiencing extrapyramidal symptoms (e.g., spasms, tremor, or involuntary movement), who did not have symptoms at baseline. For example, proportion of participants exceeding a total score of 3 on the Modified Simpson-Angus Scale (MSAS) [75].
  - Measured on a continuous scale, e.g., mean change from baseline or a value-at-a-time on the Involuntary Movement Scale, Condensed User’s Scale, Simpson-Angus Scale, or Barnes Akathesia Rating Scale.
4. Cardiovascular effects
  - Mortality because of cardiac event
  - Incidence of myocardial infarction
  - QTc interval prolongation
    i. Measured on a continuous scale, e.g., mean change from baseline or a value-at-a-time
    ii. Measured categorically, e.g., proportion of participants with a prolonged QTc interval. Where possible, we will define this as ≥450 ms in men and ≥ 460 ms in women [76]
  - Blood pressure
    i. Mean change from baseline or value-at-a-time
    ii. Incidence of orthostatic hypotension (e.g., decrease in systolic BP ≥20 mm Hg or decrease in diastolic BP ≥10 mm Hg within 3 min after standing from sitting/supine position)
5. Cholesterol
  - Triglyerides measured continuously, e.g., mean change from baseline or a value-at-a-time
  - Triglyerides measured categorically, e.g., proportion of participants with ≥150 mg/dL and proportion of participants with ≥200 mg/dL
  - Total cholesterol measured continuously, e.g., mean change from baseline or a value-at-a-time
  - Total cholesterol measured categorically, e.g., proportion of participants with ≥200 mg/dL
6. Immune effects
  - Incidence of infection
  - Incidence of low neutrophil count, e.g., absolute neutrophil count (ANC) <100/ml)
7. Mania: symptoms of mania and hypomania as measured by any scale or instrument. Depending on how mania was measured and reported in the source documents, we will analyze it as a continuous outcome or a categorical outcome.
  - Worsening of symptoms, e.g., proportion of participants scoring ≥16 or ≥20 on the Young Mania Rating Scale (YMRS) [77, 78]
  - Change in symptoms of mania, e.g., mean difference from baseline on the YMRS or another depression rating scale
8. Neuroleptic malignant syndrome (an adverse reaction characterized by fever, muscle rigidity, and cognitive and autonomic abnormalities)
9. Time-to-event data related to any domains above

#### Baseline data to record

To describe the characteristics of participants in each study, we will extract demographic and clinical characteristics from each report. For the total sample, and for each group where possible, we will record the following:

1. Age
2. Sex
3. Weight
4. Self-reported race/ethnicity (percentage of non-white)
5. Drug and alcohol use
6. Study location (country and state or county)
7. Previous response or non-response to medication (e.g., quetiapine, other antipsychotics)
8. Concurrent psychiatric medication use (which will be described individually and in classes, such as antipsychotics, anticonvulsants, selective serotonin reuptake inhibitors, tricyclics, MAOIs, and lithium)
9. Duration of current episode (i.e., time since onset)
10. Number of mood episodes in the last year
11. Comorbid psychiatric conditions (e.g., proportion of people with an anxiety disorder, substance use disorder, personality disorder, or other mood disorder)

### Subgroup analysis and investigation of heterogeneity

We will summarize the characteristics of included studies and describe potential sources of clinical and methodological heterogeneity between them and their potential influence on results.

We will conduct the following subgroup analyses:

1. Sex (men versus women)
2. Type of bipolar disorder (type I compared with type II)
3. Use of other concurrent medication (e.g., quetiapine as adjunct compared with quetiapine monotherapy)
4. Daily dose (variable dose studies; fixed doses of 100–300, 301–600, 601–900, and over 900 mg)

### Searching for published literature

Databases and trial registries will be searched using the terms in Appendix 2.

## Discussion

This study is now underway. We have identified outcomes for both reviews, conducted literature searches, and begin data extraction.

### Protocol amendments

If this protocol is amended, we will record a description and the date of each amendment and we will describe these changes with the final report.

## Appendix 1

### Gabapentin for neuropathic pain searches

#### Searching for published literature

Databases will be searched using the following terms.

*PubMed*

((((((((Pain Measurement[Mesh] OR Pain[Mesh] OR pain[tiab]))

AND

((“Amputation”[Mesh] OR “Amputation Stumps”[Mesh] OR “Amputation, Traumatic”[Mesh] OR “Central Nervous System Diseases”[Mesh] OR “Diabetes Mellitus”[Mesh] OR “Diabetes Mellitus, Type 2”[Mesh] OR “Diabetes Mellitus, Type 1”[Mesh] OR “Diabetes Complications”[Mesh] OR “Guillain-Barre Syndrome”[Mesh] OR “HIV Seropositivity”[Mesh] OR “HIV Infections”[Mesh] OR “Acquired Immunodeficiency Syndrome”[Mesh] OR “Multiple Sclerosis”[Mesh] OR “Neoplasms”[Mesh] OR “Neoplasm Recurrence, Local”[Mesh] OR “Neoplasm Metastasis”[Mesh] OR “Neoplasm Invasiveness”[Mesh] OR “Drug Resistance, Neoplasm”[Mesh] OR “Nervous System Neoplasms”[Mesh] OR “Causalgia”[Mesh] OR “Complex Regional Pain Syndromes”[Mesh] OR “Reflex Sympathetic Dystrophy”[Mesh] OR “Phantom Limb”[Mesh] OR “Trauma, Nervous System”[Mesh] OR “Spinal Cord Injuries”[Mesh] OR “Thoracic Injuries”[Mesh] OR “Stroke”[Mesh] OR “Trigeminal Neuralgia”[Mesh] OR “Carpal Tunnel Syndrome”[Mesh]) OR (amputation[tiab] OR amputations[tiab] OR traumatic[tiab] OR trauma[tiab] OR diabetes[tiab] OR GBS[tiab] OR “demyelinating polyneuropathy”[tiab] OR “demyelinating polyneuropathies”[tiab] OR “Acute Autoimmune Neuropathy”[tiab] OR “acute autoimmune neuropathies”[tiab] OR “acute inflammatory polyneuropathy”[tiab] OR “acute inflammatory polyneuropathies”[tiab] OR “guillain barre syndrome”[tiab] OR “guillaine barre syndrome”[tiab] OR “guillainbarre syndrome”[tiab] OR HIV[tiab] OR AIDS[tiab] OR “human immunodeficiency virus”[tiab] OR “multiple sclerosis”[tiab] OR tumor[tiab] OR tumour[tiab] OR cancer[tiab] OR malignancy[tiab] OR carcinoma[tiab] OR neoplasm[tiab] OR tumors[tiab] OR tumours[tiab] OR cancers[tiab] OR malignancies[tiab] OR carcinomas[tiab] OR neoplasms[tiab] OR neuralgia[tiab] OR neuralgias[tiab] OR “phantom limb pain”[tiab] OR “phantom limb pains”[tiab] OR “complex regional pain syndrome”[tiab] OR “complex regional pain syndromes”[tiab] OR CRPS[tiab] OR causalgia[tiab] OR “spinal cord injury”[tiab] OR stroke[tiab] OR strokes[tiab] OR paralysis[tiab] OR “reflex sympathetic dystrophy”[tiab] OR “tic douloureux”[tiab] OR “carpal tunnel syndrome”[tiab])))) OR ((“Paresthesia”[Mesh] OR “Somatosensory Disorders”[Mesh] OR “Neuralgia”[Mesh] OR “Polyradiculoneuropathy”[Mesh] OR “Paraneoplastic Polyneuropathy”[Mesh] OR “Peripheral Nervous System Diseases/drug therapy”[Majr]) OR (paresthesia[tiab] OR paraesthesia[tiab] OR paresthesias[tiab] OR paraesthesias[tiab] OR neuropathic pain[tiab] OR painful neuropathy[tiab] OR neuropathic chronic pain[tiab]))) OR ((Diabetic Neuropathies[Mesh] OR “Herpes Zoster/complications”[Mesh] OR “Neuralgia, Postherpetic”[Mesh]) OR (“diabetes complication”[tiab] OR “diabetes complications”[tiab] OR “diabetic complication”[tiab] OR “diabetic complications”[tiab] OR “diabetic neuropathy”[tiab] OR PDN[tiab] OR “diabetic neuropathies”[tiab] OR “diabetic polyneuropathy”[tiab] OR “diabetic polyneuropathies”[tiab] OR “diabetic autonomic neuropathy”[tiab] OR “diabetic autonomic neuropathies”[tiab] OR “diabetic mononeuropathy”[tiab] OR “diabetic mononeuropathies”[tiab] OR “diabetic amyotrophy”[tiab] OR herpes[tiab] OR shingles[tiab] OR zoster[tiab] OR HSV[tiab] OR “diabetic neuropathic pain”[tiab] OR DPN[tiab] OR PHN[tiab] OR “neuropathic cancer pain”[tiab] OR “chemotherapy-induced peripheral neuropathy”[tiab] OR CINP[tiab]))))

AND

((Amines[Mesh] OR “Cyclohexanecarboxylic Acids”[Mesh] OR “gamma-Aminobutyric Acid”[Mesh:noexp] OR “GABA Agonists”[Mesh] OR “GABA Agents”[Mesh] OR “Anticonvulsants”[Mesh] OR “Anticonvulsants”[Pharmacological Action] OR Epilepsy/drug effects[Mesh] OR “Epilepsy/drug therapy”[Mesh] OR “Epilepsy/therapy”[Mesh]) OR (anticonvulsant[tiab] OR anticonvulsants[tiab] OR “anti-convulsant”[tiab] OR “anti-convulsants”[tiab] OR “antiepileptic drug”[tiab] OR “antiepileptic drugs”[tiab] OR “anti-epileptic drug”[tiab] OR “anti-epileptic drugs”[tiab] OR “antiseizure drug”[tiab] OR “antiseizure drugs”[tiab] OR “anti-seizure drug”[tiab] OR “anti-seizure drugs”[tiab] OR “antiseizure agent”[tiab] OR “antiseizure agents”[tiab] OR “anti-seizure agent”[tiab] OR “anti-seizure agents”[tiab] OR “antiepileptic agent”[tiab] OR “antiepileptic agents”[tiab] OR “anti-epileptic agent”[tiab] OR “anti-epileptic agents”[tiab] OR gabapentin[tiab] OR Neurontin[tiab])))

AND

(randomized controlled trial[pt] OR controlled clinical trial[pt] OR randomized[tiab] OR placebo[tiab] OR clinical trials as topic[mesh:noexp] OR randomly[tiab] OR trial[ti]) NOT (Animals[mesh] NOT humans[mesh])

*Embase.com*

#### Combination of concepts

#1 Pain AND Conditions

#2 Neuropathic pain OR Condition-specific neuropathic pain

#3 Drugs

#4 Cochrane UK RCT Terms

#5 (#1 OR #2) AND #3 AND #4

#### Pain

“pain”/exp OR “chronic pain”/exp OR “pain assessment”/exp OR “neuralgia”/exp OR “visual analog scale”/exp OR pain:ab,ti OR “visual analog scale”:ab,ti OR “vas”:ab,ti OR “visual analogue scale”:ab,ti OR neuralgia:ab,ti

#### Neuropathic pain

paresthesia”/exp OR “neuralgia”/exp OR “neuropathic pain”/exp OR “somatosensory disorder”/exp OR “polyneuropathy”/exp OR “polyradiculoneuropathy”/exp OR “peripheral nerve injury”/exp OR “neuropathy”/exp OR “dysesthesia”/exp OR “peripheral neuropathy”/exp OR “sensory neuropathy”/exp OR “sensory dysfunction”/exp OR neuralgia:ab,ti OR neuralgias:ab,ti OR “neuropathic pain”:ab,ti OR paresthesia:ab,ti OR paraesthesia:ab,ti OR paresthesias:ab,ti OR paraesthesias:ab,ti OR polyneuropathy:ab,ti OR polyneuropathies:ab,ti OR polyradiculoneuropathy:ab,ti OR polyradiculoneuropathies:ab,ti OR dysesthesia:ab,ti OR dysesthesias:ab,ti

#### Conditions

“radicular pain”/exp OR “radiculopathy”/exp OR “low back pain”/exp OR “diabetes mellitus”/de OR “non insulin dependent diabetes mellitus”/exp OR “guillain barre syndrome”/exp OR “herpes zoster”/exp OR “human immunodeficiency virus”/de OR “human immunodeficiency virus infection”/de OR “multiple sclerosis”/exp OR “malignant neoplastic disease”/exp OR “cancer pain”/exp OR “postoperative pain”/exp OR “sensory neuropathy”/exp OR “peripheral nerve injury”/exp OR “causalgia”/exp OR “complex regional pain syndrome type 1”/exp OR “spinal cord injury”/exp OR “vertebral canal stenosis”/exp OR “intermittent claudication”/exp OR “nervous system injury”/exp OR “stroke”/exp OR “paraplegia”/exp OR “phantom pain”/exp OR “amputation”/exp OR “carpal tunnel syndrome”/exp OR “trigeminus neuralgia”/exp OR amputation:ab,ti OR amputations:ab,ti OR “radicular pain”:ab,ti OR radiculopathy:ab,ti OR radiculopathies:ab,ti OR “low back pain”:ab,ti OR “lower back pain”:ab,ti OR diabetes:ab,ti OR “guillain-barre syndrome”:ab,ti OR “guillainbarre syndrome”:ab,ti OR “guillaine barre syndrome”:ab,ti OR gbs:ab,ti OR “demyelinating polyneuropathy”:ab,ti OR “demyelinating polyneuropathies”:ab,ti OR “acute autoimmune neuropathy”:ab,ti OR “acute autoimmune neuropathies”:ab,ti OR “acute inflammatory polyneuropathy”:ab,ti OR “acute inflammatory polyneuropathies”:ab,ti OR herpes:ab,ti OR shingles:ab,ti OR zoster:ab,ti OR hsv:ab,ti OR hiv:ab,ti OR aids:ab,ti OR “human immunodeficiency virus”:ab,ti OR “multiple sclerosis”:ab,ti OR cancer:ab,ti OR tumor:ab,ti OR tumour:ab,ti OR malignancy:ab,ti OR carcinoma:ab,ti OR neoplasm:ab,ti OR tumors:ab,ti OR tumours:ab,ti OR cancers:ab,ti OR malignancies:ab,ti OR carcinomas:ab,ti OR neoplasms:ab,ti OR “phantom limb pain”:ab,ti OR “complex regional pain syndrome”:ab,ti OR crps:ab,ti OR causalgia:ab,ti OR “spinal cord injury”:ab,ti OR stroke:ab,ti OR strokes:ab,ti OR paralysis:ab,ti OR “carpal tunnel syndrome”:ab,ti OR “trigeminal neuralgia”:ab,ti OR “tic douloureux”:ab,ti

#### Condition-specific neuropathic pain concept

“diabetic neuropathy”/exp OR “postherpetic neuralgia”/exp OR “chemotherapy-induced peripheral neuropathy” OR “neuropathic cancer pain” OR “cancer pain”/exp OR “chronic post-thoractomy pain” OR “phantom pain”/exp OR “posttraumatic pain”/exp OR “postoperative pain”/exp OR “diabetes complication”:ab,ti OR “diabetes complications”:ab,ti OR “diabetic complication”:ab,ti OR “diabetic complications”:ab,ti OR “diabetic neuropathy”:ab,ti OR “diabetic neuropathies”:ab,ti OR “diabetic neuralgia”:ab,ti OR “diabetic neuralgias”:ab,ti OR pdn:ab,ti OR dpn:ab,ti OR “diabetic polyneuropathy”:ab,ti OR “diabetic polyneuropathies”:ab,ti OR “diabetic autonomic neuropathy”:ab,ti OR “diabetic autonomic neuropathies”:ab,ti OR “diabetic asymmetric polyneuropathy”:ab,ti OR “diabetic asymmetric polyneuropathies”:ab,ti OR “diabetic mononeuropathy”:ab,ti OR “diabetic mononeuropathies”:ab,ti OR “diabetic amyotrophy”:ab,ti OR “post-herpetic neuralgia”:ab,ti OR “post-herpetic neuralgias”:ab,ti OR “postherpetic neuralgia”:ab,ti OR “postherpetic neuralgias”:ab,ti OR “post-herpetic neuropathic pain”:ab,ti OR “postherpetic neuropathic pain”:ab,ti OR (“chemotherapy”:ab,ti AND “neuropathic pain”:ab,ti) OR “cancer pain”:ab,ti OR (“chronic”:ab,ti AND (“postoperative”:ab,ti OR “post-operative”:ab,ti OR “post operative”:ab,ti OR “postsurgical”:ab,ti OR “post-surgical”:ab,ti) AND “pain”:ab,ti)

#### Drug term

“4 aminobutyric acid”/de OR “cyclohexanecarboxylic acid derivative”/exp OR “gabapentin”/exp OR “gabapentin enacarbil”/exp OR “anticonvulsive agent”/de OR anticonvulsant:ab,ti OR anticonvulsants:ab,ti OR “anti-convulsant”:ab,ti OR “anti-convulsants”:ab,ti OR “antiepileptic drug”:ab,ti OR “antiepileptic drugs”:ab,ti OR “anti-epileptic drug”:ab,ti OR “anti-epileptic drugs”:ab,ti OR “antiseizure drug”:ab,ti OR “antiseizure drugs”:ab,ti OR “anti-seizure drug”:ab,ti OR “anti-seizure drugs”:ab,ti OR “antiseizure agent”:ab,ti OR “antiseizure agents”:ab,ti OR “anti-seizure agent”:ab,ti OR “anti-seizure agents”:ab,ti OR “antiepileptic agent”:ab,ti OR “antiepileptic agents”:ab,ti OR “anti-epileptic agent”:ab,ti OR “anti-epileptic agents”:ab,ti OR gabapentin:ab,ti OR Neurontin:ab,ti

#### Cochrane UK RCT Terms for Embase

random* OR factorial* OR crossover* OR cross NEXT/1 over* OR placebo* OR doubl* NEXT/1 blind* OR singl* NEXT/1 blind* OR assign* OR allocate* OR volunteer* OR “crossover procedure”/exp OR “double blind procedure”/exp OR “randomized controlled trial”/exp OR “single blind procedure”/exp

*Central*

#1 MeSH descriptor: [Pain] explode all trees

#2 MeSH descriptor: [Pain Measurement] explode all trees

#3 “pain” (Word variations have been searched)

#4 #1 or #2 or #3

#5 MeSH descriptor: [Amputation] explode all trees

#6 MeSH descriptor: [Amputation Stumps] explode all trees

#7 MeSH descriptor: [Amputation, Traumatic] explode all trees

#8 MeSH descriptor: [Central Nervous System Diseases] explode all trees

#9 MeSH descriptor: [Diabetes Mellitus] explode all trees

#10 MeSH descriptor: [Diabetes Complications] explode all trees

#11 MeSH descriptor: [Guillain-Barre Syndrome] explode all trees

#12 MeSH descriptor: [HIV Seropositivity] explode all trees

#13 MeSH descriptor: [HIV Infections] explode all trees

#14 MeSH descriptor: [Acquired Immunodeficiency Syndrome] explode all trees

#15 MeSH descriptor: [Multiple Sclerosis] explode all trees

#16 MeSH descriptor: [Neoplasms] explode all trees

#17 MeSH descriptor: [Neoplasm Recurrence, Local] explode all trees

#18 MeSH descriptor: [Neoplasm Metastasis] explode all trees

#19 MeSH descriptor: [Neoplasm Invasiveness] explode all trees

#20 MeSH descriptor: [Drug Resistance, Neoplasm] explode all trees

#21 MeSH descriptor: [Nervous System Neoplasms] explode all trees

#22 MeSH descriptor: [Causalgia] explode all trees

#23 MeSH descriptor: [Complex Regional Pain Syndromes] explode all trees

#24 MeSH descriptor: [Reflex Sympathetic Dystrophy] explode all trees

#25 MeSH descriptor: [Phantom Limb] explode all trees

#26 MeSH descriptor: [Trauma, Nervous System] explode all trees

#27 MeSH descriptor: [Spinal Cord Injuries] explode all trees

#28 MeSH descriptor: [Thoracic Injuries] explode all trees

#29 MeSH descriptor: [Stroke] explode all trees

#30 MeSH descriptor: [Trigeminal Neuralgia] explode all trees

#31 MeSH descriptor: [Carpal Tunnel Syndrome] explode all trees

#32 (amputation or amputations or traumatic or trauma or diabetes or GBS or “demyelinating polyneuropathy” or “demyelinating polyneuropathies” or “Acute Autoimmune Neuropathy” or “acute autoimmune neuropathies” or “acute inflammatory polyneuropathy” or “acute inflammatory polyneuropathies” or “guillain barre syndrome” or “guillaine barre syndrome” or “guillainbarre syndrome” or HIV or AIDS or “human immunodeficiency virus” or “multiple sclerosis” or tumor or tumour or cancer or malignancy or carcinoma or neoplasm or tumors or tumours or cancers or malignancies or carcinomas or neoplasms or neuralgia or neuralgias or “phantom limb pain” or “phantom limb pains” or “complex regional pain syndrome” or “complex regional pain syndromes” or CRPS or causalgia or “spinal cord injury” or stroke or strokes or paralysis or “reflex sympathetic dystrophy” or “reflex sympathetic dystrophies” or “tic douloureux” or “carpal tunnel syndrome”) (Word variations have been searched)

#33 #5 or #6 or #7 or #8 or #9 or #10 or #11 or #12 or #13 or #14 or #15 or #16 or #17 or #18 or #19 or #20 or #21 or #22 or #23 or #24 or #25 or #26 or #27 or #28 or #29 or #30 or #31 or #32

#34 #4 and #33

#35 MeSH descriptor: [Paresthesia] explode all trees

#36 MeSH descriptor: [Somatosensory Disorders] explode all trees

#37 MeSH descriptor: [Neuralgia] explode all trees

#38 MeSH descriptor: [Polyradiculoneuropathy] explode all trees

#39 MeSH descriptor: [Paraneoplastic Polyneuropathy] explode all trees

#40 MeSH descriptor: [Peripheral Nervous System Diseases] explode all trees and with qualifier(s): [Drug therapy - DT]

#41 (paresthesia or paraesthesia or paresthesias or paraesthesias or neuropathic pain or painful neuropathy or neuropathic chronic pain) (Word variations have been searched)

#42 #35 or #36 or #37 or #38 or #39 or #40 or #41

#43 MeSH descriptor: [Diabetic Neuropathies] explode all trees

#44 MeSH descriptor: [Herpes Zoster] explode all trees and with qualifier(s): [Complications - CO]

#45 MeSH descriptor: [Neuralgia, Postherpetic] explode all trees

#46 (“diabetes complication” or “diabetes complications” or “diabetic complication” or “diabetic complications” or “diabetic neuropathy” or PDN or “diabetic neuropathies” or “diabetic polyneuropathy” or “diabetic polyneuropathies” or “diabetic autonomic neuropathy” or “diabetic autonomic neuropathies” or “diabetic mononeuropathy” or “diabetic mononeuropathies” or “diabetic amyotrophy” or herpes or shingles or zoster or HSV or “diabetic neuropathic pain” or DPN or PHN or “neuropathic cancer pain” or “chemotherapy-induced peripheral neuropathy” or CINP) (Word variations have been searched)

#47 #43 or #44 or #45 or #46

#48 #34 or #42 or #47

#49 MeSH descriptor: [Amines] explode all trees

#50 MeSH descriptor: [Cyclohexanecarboxylic Acids] explode all trees

#51 MeSH descriptor: [gamma-Aminobutyric Acid] this term only

#52 MeSH descriptor: [GABA Agonists] explode all trees

#53 MeSH descriptor: [GABA Agents] explode all trees

#54 MeSH descriptor: [Anticonvulsants] explode all trees

#55 MeSH descriptor: [Epilepsy] explode all trees

#56 (anticonvulsant or anticonvulsants or “anti-convulsant” or “anti-convulsants” or “antiepileptic drug” or “antiepileptic drugs” or “anti-epileptic drug” or “anti-epileptic drugs” or “antiseizure drug” or “antiseizure drugs” or “anti-seizure drug” or “anti-seizure drugs” or “antiseizure agent” or “antiseizure agents” or “anti-seizure agent” or “anti-seizure agents” or “antiepileptic agent” or “antiepileptic agents” or “anti-epileptic agent” or “anti-epileptic agents” or gabapentin or Neurontin) (Word variations have been searched)

#57 #49 or #50 or #51 or #52 or #53 or #54 or #55 or #56

#58 #48 and #57

*CINAHL (Ebsco)*

#### Combination of concepts

#1 Pain AND Conditions

#2 Neuropathic pain OR Condition-specific neuropathic pain

#3 Drugs

#5 (#1 OR #2) AND #3

Pain concept

(MH “Pain”) OR (MH “Back Pain”) OR (MH “Low Back Pain”) OR (MH “Facial Pain”) OR (TI pain OR AB pain)

#### Neuropathic pain concept

((MH “Paresthesia+“) OR (MH “Somatosensory Disorders+“) OR (MH “Neuralgia+”) OR (MH “Polyneuropathies+”) OR (MH “Neuralgia+”)) OR (TI paresthesia OR AB paresthesia OR TI paraesthesia OR AB paraesthesia OR TI paresthesias OR AB paresthesias OR TI paraesthesias OR AB paraesthesias OR TI neuropathic pain OR AB neuropathic pain OR TI painful neuropathy OR AB painful neuropathy OR TI painful neuropathies OR AB painful neuropathies OR TI neuropathic chronic pain OR AB neuropathic chronic pain OR TI Polyradiculoneuropathy OR AB Polyradiculoneuropathy OR TI Polyradiculoneuropathies OR AB Polyradiculoneuropathies OR TI neuralgia OR AB neuralgia OR TI neuralgias OR AB neuralgias)

#### Conditions concept

((MH “Amputation+”) OR (MH “Phantom Pain”) OR (MH “Amputation Stumps”) OR (MH “Amputation, Traumatic”) OR (MH “Diabetes Mellitus+”) OR (MH “Diabetes Mellitus, Non-Insulin-Dependent”) OR (MH “Diabetes Mellitus, Insulin-Dependent”) OR (MH “Guillain-Barre Syndrome”) OR (MH “HIV Seropositivity”) OR (MH “HIV Infections+“) OR (MH “Acquired Immunodeficiency Syndrome“) OR (MH “Human Immunodeficiency Virus+“) OR (MH “Multiple Sclerosis“) OR (MH “Neoplasms+“) OR (MH “Carcinoma+”) OR (MH “Neoplasm Recurrence, Local”) OR (MH “Neoplasm Metastasis”) OR (MH “Neoplasm Invasiveness”) OR (MH “Causalgia”) OR (MH “Complex Regional Pain Syndromes”) OR (MH “Reflex Sympathetic Dystrophy”) OR (MH “Phantom Limb”) OR (MH “Spinal Cord Injuries+”) OR (MH “Stroke”) OR (MH “Trauma+”) OR (MH “Trigeminal Neuralgia”) OR (MH “Low Back Pain+”)) OR (TI amputation OR AB amputation OR TI amputations OR AB amputations OR TI traumatic OR AB traumatic OR TI trauma OR AB trauma OR TI diabetes OR AB diabetes OR TI “guillain barre syndrome” OR AB “guillain barre syndrome” OR TI “guillaine barre syndrome” OR AB “guillaine barre syndrome” OR TI “guillainbarre syndrome” OR AB “guillainbarre syndrome” OR TI GBS OR AB GBS OR TI “demyelinating polyneuropathy” OR AB “demyelinating polyneuropathy” OR TI “demyelinating polyneuropathies” OR AB “demyelinating polyneuropathies” OR TI “Acute Autoimmune Neuropathy” OR AB “Acute Autoimmune Neuropathy” OR TI “acute autoimmune neuropathies” OR AB “acute autoimmune neuropathies” OR TI “acute inflammatory polyneuropathy” OR AB “acute inflammatory polyneuropathy” OR TI “acute inflammatory polyneuropathies” OR AB “acute inflammatory polyneuropathies” OR TI HIV OR AB HIV OR TI AIDS OR AB AIDS OR TI “human immunodeficiency virus” OR AB “human immunodeficiency virus” OR TI “multiple sclerosis” OR AB “multiple sclerosis” OR TI tumor OR AB tumor OR TI tumour OR AB tumour OR TI cancer OR AB cancer OR TI malignancy OR AB malignancy OR TI carcinoma OR AB carcinoma OR TI tumors OR AB tumors OR TI tumours OR AB tumours OR TI cancers OR AB cancers OR TI malignancies OR AB malignancies OR TI carcinomas OR AB carcinomas OR TI “phantom limb pain” OR AB “phantom limb pain” OR TI “complex regional pain syndrome” OR AB “complex regional pain syndrome” OR TI “complex regional pain syndromes” OR AB “complex regional pain syndromes” OR TI CRPS OR AB CRPS OR TI causalgia OR AB causalgia OR TI causalgias OR AB causalgias OR TI “spinal cord injury” OR AB “spinal cord injury” OR TI “spinal cord injuries” OR AB “spinal cord injuries” OR TI stroke OR AB stroke OR TI strokes OR AB strokes OR TI paralysis OR AB paralysis OR TI “reflex sympathetic dystrophy” OR AB “reflex sympathetic dystrophy” OR TI “reflex sympathetic dystrophies” OR AB “reflex sympathetic dystrophies” OR TI “trigeminal neuralgia” OR AB “trigeminal neuralgia” OR TI “trigeminal neuralgias” OR AB “trigeminal neuralgias” OR TI “tic doloreux” OR AB “tic doloreux” OR TI “Diabetes Complications” OR AB “Diabetes Complications”)

#### Condition-specific neuropathic pain concept

((MH “Diabetic Neuropathies+“) OR (MH “Herpes Zoster+/CO”) OR (MH “Neuralgia, Postherpetic+“) OR (MH “Cancer Pain+”)) OR (TI “diabetes complication” OR AB “diabetes complication” OR TI “diabetic complication” OR AB “diabetic complication” OR TI “diabetic complications” OR AB “diabetic complications” OR TI “painful diabetic neuropathy” OR AB “painful diabetic neuropathy” OR TI “diabetic neuralgia” OR AB “diabetic neuralgia” OR TI PDN OR AB PDN OR TI “diabetic neuropathies” OR AB “diabetic neuropathies” OR TI “diabetic polyneuropathy” OR AB “diabetic polyneuropathy” OR TI “diabetic polyneuropathies” OR AB “diabetic polyneuropathies” OR TI “diabetic autonomic neuropathy” OR AB “diabetic autonomic neuropathy” OR TI “diabetic mononeuropathy” OR AB “diabetic mononeuropathy” OR TI “diabetic mononeuropathies” OR AB “diabetic mononeuropathies” OR TI “diabetic amyotrophy” OR AB “diabetic amyotrophy” OR TI herpes OR AB herpes OR TI shingles OR AB shingles OR TI zoster OR AB zoster OR TI HSV OR AB HSV OR TI “diabetic neuropathic pain” OR AB “diabetic neuropathic pain” OR TI DPN “ OR AB DPN OR TI PHN OR AB PHN OR TI “neuropathic cancer pain” OR AB “neuropathic cancer pain” OR TI “neuropathic pain syndrome” OR AB “neuropathic pain syndrome” OR TI “neuropathic pain syndromes” OR AB “neuropathic pain syndromes” OR TI “chemotherapy-induced neuropathic pain” OR AB “chemotherapy-induced neuropathic pain” OR TI “chemotherapy-induced peripheral neuropathy” OR AB “chemotherapy-induced peripheral neuropathy” OR TI “chemotherapy induced neuropathic pain “ OR AB “chemotherapy induced neuropathic pain” OR TI CINP OR AB CINP)

#### Drugs concept for gabapentin alone

((MH “GABA”) OR (MH “GABA Agonists+“) OR (MH “GABA Agents+”) OR (MH “Anticonvulsants+“) OR (MH “Epilepsy+/DT/TH”) OR (MH “Gabapentin”)) OR (TI anticonvulsant OR AB anticonvulsant OR TI anticonvulsants OR AB anticonvulsants OR TI “anti-convulsant” OR AB “anti-convulsant” OR TI “anti-convulsants” OR AB “anti-convulsants” OR TI “antiepileptic drug” OR AB “antiepileptic drug” OR TI “antiepileptic drugs” OR AB “antiepileptic drugs” OR TI “anti-epileptic drug” OR AB “anti-epileptic drug” OR TI “anti-epileptic drugs” OR AB “anti-epileptic drugs” OR TI “antiseizure drug” OR AB “antiseizure drug” OR TI “antiseizure drugs” OR AB “antiseizure drugs” OR TI “anti-seizure agent” OR AB “anti-seizure agent” OR TI “anti-seizure agents” OR AB “anti-seizure agents” OR TI “antiepileptic agent” OR AB “antiepileptic agent” OR TI “antiepileptic agents” OR AB “antiepileptic agents” OR TI “anti-epileptic agent” OR AB “anti-epileptic agent” OR TI “anti-epileptic agents” OR AB “anti-epileptic agents” OR TI gabapentin OR AB gabapentin OR TI Neurontin OR AB Neurontin)

*LILACS*

(((pain OR C10.597.617$ OR C23.888.646$ OR E01.370.600.550.324) AND (amputation OR amputations OR E04.555.080$ OR traumatic OR trauma OR diabetes OR “guillain-barre syndrome” OR “guillaine-barre syndrome” OR “guillaine barre syndrome” OR “guillain barre syndrome” OR “guillainbarre syndrome” OR C10.114.750$ OR GBS OR “demyelinating polyneuropathy” OR “demyelinating polyneuropathies” OR C10.314$ OR C10.668.829.800$ OR “Acute Autoimmune Neuropathy” OR “acute autoimmune neuropathies” OR “acute inflammatory polyneuropathy” OR “acute inflammatory polyneuropathies” OR HIV OR AIDS OR “human immunodeficiency virus” OR C20.673.480$ OR “multiple sclerosis” OR tumor OR tumour OR cancer OR malignancy OR carcinoma OR tumors OR tumours OR cancers OR malignancies OR carcinomas OR C04$ OR neuralgia OR neuralgias OR “phantom limb pain” OR “complex regional pain syndrome” OR “complex regional pain syndromes” OR CRPS OR causalgia OR causalgias OR C10.177.195$ OR “spinal cord injury” OR “spinal cord injuries” OR C26.819$ OR C10.900.850$ OR stroke OR strokes OR C10.228.140.300.775$ OR paralysis OR C23.888.592.636$ OR “reflex sympathetic dystrophy” OR “reflex sympathetic dystrophies”)) OR (paresthesia OR paraesthesia OR paresthesias OR paraesthesias OR C10.597.751.791.875 OR “neuropathic pain” OR “painful neuropathy” OR “neuropathic chronic pain” OR Neuropatías OR Neuropatias OR Polineuropatía OR Polineuropatia OR C10.668.829.500$ OR C10.668.829$) OR (“diabetes complication” OR “diabetes complications” OR “diabetic complication” OR “diabetic complications” OR “diabetic neuropathy” OR “diabetic neuropathies” OR “diabetic neuralgia” OR “diabetic neuralgias” OR PDN OR “diabetic polyneuropathy” OR “diabetic polyneuropathies” OR “diabetic autonomic neuropathy” OR “diabetic autonomic neuropathies” OR “diabetic mononeuropathy” OR “diabetic mononeuropathies” OR “diabetic amyotrophy” OR C19.246.099.937$ OR herpes OR shingles OR zoster OR HSV OR “neuropathic diabetic pain” OR DPN OR PHN OR “neuropathic cancer pain” OR “neuropathic pain syndrome” OR “neuropathic pain syndromes” OR “neuropathic chemotherapy-induced pain” OR “neuropathic chemotherapy induced pain” OR “chemotherapy-induced peripheral neuropathy” OR “chemotherapy induced peripheral neuropathy” OR “chemotherapy-induced peripheral neuropathies” OR “chemotherapy induced peripheral neuropathies” OR CINP)) AND (anticonvulsant OR anticonvulsants OR “anti-convulsant” OR “anti-convulsants” OR “anti convulsant” OR “anti convulsants” OR D27.505.954.427.080 OR “antiepileptic drug” OR “antiepileptic drugs” OR “anti-epileptic drug” OR “anti-epileptic drugs” OR “anti epileptic drug” OR “anti epileptic drugs” OR “antiseizure drug” OR “antiseizure drugs” OR “anti-seizure drug” OR “anti-seizure drugs” OR “anti seizure drug” OR “anti seizure drugs” OR “antiseizure agent” OR “antiseizure agents” OR “anti-seizure agent” OR “anti-seizure agents” OR “anti seizure agent” OR “anti seizure agents” OR “antiepileptic agent” OR “antiepileptic agents” OR “anti-epileptic agent” OR “anti-epileptic agents” OR “anti epileptic agent” OR “anti epileptic agents” OR gabapentin OR Neurontin OR D27.505.519.625.240$ OR mh:”Neuralgia/therapy” OR mh:”Neuralgia/ uso terapéutico”)

### Searching trial registries

We will conduct a basic search of ClinicaTrials.gov using the terms below. We will conduct a standard search of the ICTRP Search Portal using the same terms, and we will remove the records from ClinicaTrials.gov from these results before screening.

“945 OR Aclonium OR Alpentin OR Anabix OR Aneptir OR Apentin OR Apo-Gab OR Bapex OR Blugat OR Calmpent OR Compulxine OR Dineurin OR Epiven OR Epleptin OR Equipax OR Gabacalma OR Gabacross OR Gabadoz OR Gabagamma OR Gabahexal OR Gabalept OR GabaLich OR Gabalix OR Gabamed OR Gabamerck OR Gabamox OR Gabaneurin OR Gabanicht OR Gabanox OR Gabantine OR Gabantin OR Gabapentin OR Gabaran OR Gabaratio OR Gabarex OR Gabarone OR Gabaseis OR Gabatal OR Gabatem OR Gabateva OR Gabatifin OR Gabatine OR Gabatin OR Gabator OR Gabatur OR Gabax OR Gabenta OR Gabental OR Gabexal OR Gabex OR Gabexine OR Gabictal OR Gabin OR Gabix OR Gabmylan OR Gabrion OR Gabtin OR Gabture OR Galepsi OR Ganin OR Gantin OR Gapenten OR Gapridol OR Geabatan OR Gordius OR Gralise OR Grimodin OR Mengaptrix OR Molnarux OR Nepatic OR Neruda OR Neugabin OR Neuran OR Neurexal OR Neuril OR Neuroba OR Neurontin OR Neuros OR Neurostil OR Nopatic OR Normatol OR Nupentin OR Nurabax OR Oxaquin OR Pendine OR Pentagab OR Pentin OR Peronten OR Progresse OR Reinin OR Ritmenal OR Semerial OR Seni-Ven OR Sipentin OR Sympleptic OR Tebantin OR Therapentin OR Tremepen OR Vultin OR Yalipent”

## Appendix 2

### Quetiapine for bipolar depression searches

#### Searching for published literature

Databases will be searched using the following terms.

*Medline (OVID)*

1. Randomized Controlled Trial.pt.
2. Controlled Clinical Trial.pt.
3. (randomized or randomised).ab,ti.
4. placebo.ab,ti.
5. drug therapy.fs.
6. randomly.ab,ti.
7. trial.ab,ti.
8. groups.ab,ti.
9. 1 or 2 or 3 or 4 or 5 or 6 or 7 or 8
10. exp animals/ not humans.sh.
11. 9 not 10
12. exp Bipolar Disorder/ or Mood Disorders/
13. (Bipolar or “bi polar”).tw.
14. ((rapid or ultradian) adj5 cycl*).tw.
15. (cyclothymic* or hypomani* or mania* or manic* or mixed episode* or RCBD).tw.
16. ((affective* or mood) adj (disorder* or disturbance* or dysfunction* or illness* or swing*)).tw.
17. 12 or 13 or 14 or 15 or 16
18. exp Dibenzothiazepines/
19. (quetiapine or Seroquel or “ICI 204636” or “ICI 204646” or ICI204636 or ICI204646 or Socalm or Tienapine or Alzen or Asicot or Bonogren or Cedrina or Cizyapine or Derin or Equepin or Equeta or Etiagen or Etipin or Geldoren or Generiapin or Gentiapin or Gofyl or Gyrex or Hedonin or Ilufren or Kefrenex or Ketidose or Ketilept or Ketinel or Ketipinor or Ketiron or Ketrel or Ketya or Kventiax or Kwetaplex or Kwetinor or Lantiapin or Lobarr or Loplive or Loquen or Mylaquel or Nantarid or Neurorace or Neutapin or Notiabolfen or Pinapaz or Poetra or Psicotric or QiWei or Qudix or Quelan or Quelor or Quemed or Quemyl or Quentapil or Quentiax or Quepin or Quepita or Quepsan or Questax or Quetapel or Quetastad or Queteper or Quetex or Quetiafair or Quetialan or Quetiamylan or Quetiazic or Quetifi or Quetilis or Quetin or Quetiratio or Quetiron or Quetiser or Quetkare or Quetrop or Qurax or Qutipin or Q-win or Raikar or Resirentin or Rocoz or Seotiapim or Sequa or Sequase or Seratio or Seresano or Serex or Seronia or Seropia or Seroquin or Setinin or “Shu Si” or Sondate or Stadaquel or Symquel or Syquet or Taiqutabs or Tevaquel or Tiaquin or “Tim ASF” or Viketo or Vorta or Xeroquel or norquetiapine).mp.
20. 18 or 19
21. 17 and 20
22. 11 and 21

*Embase.com*

#1 “randomized controlled trial”/exp

#2 “randomization”/exp

#3 “double blind procedure”/exp

#4 “single blind procedure”/exp

#5 random*:ab,ti

#6 #1 OR #2 OR #3 OR #4 OR #5

#7 “animal”/exp OR “animal experiment”/exp

#8 “human”/exp

#9 #7 AND #8

#10 #7 NOT #9

#11 #6 NOT #10

#12 “clinical trial”/exp

#13 (clin* NEAR/3 trial*):ab,ti

#14 ((singl* OR doubl* OR trebl* OR tripl*) NEAR/3 (blind* OR mask*)):ab,ti

#15 “placebo”/exp

#16 placebo*:ab,ti

#17 random*:ab,ti

#18 “experimental design”/exp

#19 “crossover procedure”/exp

#20 “control group”/exp

#21 “latin square design”/exp

#22 #12 OR #13 OR #14 OR #15 OR #16 OR #17 OR #18 OR #19 OR #20 OR #21

#23 #22 NOT #10

#24 #23 NOT #11

#25 “comparative study”/exp

#26 “evaluation”/exp

#27 “prospective study”/exp

#28 control*:ab,ti OR prospectiv*:ab,ti OR volunteer*:ab,ti

#29 #25 OR #26 OR #27 OR #28

#30 #29 NOT #10

#31 #30 NOT (#11 OR #23)

#32 #11 OR #24 OR #31

#33 “bipolar disorder”/exp OR “mania”/de OR “mood disorder”/de

#34 bipolar:ab,ti OR “bi polar”:ab,ti OR hypomani*:ab,ti OR mania*:ab,ti OR manic*:ab,ti OR rcbd:ab,ti OR cyclophren*:ab,ti OR cyclothymi*:ab,ti OR “maniodepressive”:ab,ti OR “mano depressive”:ab,ti OR (mixed NEAR/1 episode*):ab,ti

#35 ((rapid OR ultradian) NEAR/5 cycl*):ab,ti

#36 ((affective* OR mood) NEAR/1 (disorder* OR disturbance* OR dysfunction* OR illness* OR swing*)):ab,ti

#37 #33 OR #34 OR #35 OR #36

#38 “quetiapine”/exp OR “dibenzothiazepine derivative”/exp

#39 quetiapine:tn,ab,ti OR seroquel:tn,ab,ti OR “ici 204,636”:tn,ab,ti OR “ici 204636”:tn,ab,ti OR “ici 204646”:tn,ab,ti OR ici204636:tn,ab,ti OR ici204646:tn,ab,ti OR socalm:tn,ab,ti OR tienapine:tn,ab,ti OR alzen:tn,ab,ti OR asicot:tn,ab,ti OR bonogren:tn,ab,ti OR cedrina:tn,ab,ti OR cizyapine:tn,ab,ti OR derin:tn,ab,ti OR equepin:tn,ab,ti OR equeta:tn,ab,ti OR etiagen:tn,ab,ti OR etipin:tn,ab,ti OR geldoren:tn,ab,ti OR generiapin:tn,ab,ti OR gentiapin:tn,ab,ti OR gofyl:tn,ab,ti OR gyrex:tn,ab,ti OR hedonin:tn,ab,ti OR ilufren:tn,ab,ti OR kefrenex:tn,ab,ti OR ketidose:tn,ab,ti OR ketilept:tn,ab,ti OR ketinel:tn,ab,ti OR ketipinor:tn,ab,ti OR ketiron:tn,ab,ti OR ketrel:tn,ab,ti OR ketya:tn,ab,ti OR kventiax:tn,ab,ti OR kwetaplex:tn,ab,ti OR kwetinor:tn,ab,ti OR lantiapin:tn,ab,ti OR lobarr:tn,ab,ti OR loplive:tn,ab,ti OR loquen:tn,ab,ti OR mylaquel:tn,ab,ti OR nantarid:tn,ab,ti OR neurorace:tn,ab,ti OR neutapin:tn,ab,ti OR notiabolfen:tn,ab,ti OR pinapaz:tn,ab,ti OR poetra:tn,ab,ti OR psicotric:tn,ab,ti OR qiwei:tn,ab,ti OR qudix:tn,ab,ti OR quelan:tn,ab,ti OR quelor:tn,ab,ti OR quemed:tn,ab,ti OR quemyl:tn,ab,ti OR quentapil:tn,ab,ti OR quentiax:tn,ab,ti OR quepin:tn,ab,ti OR quepita:tn,ab,ti OR quepsan:tn,ab,ti OR questax:tn,ab,ti OR quetapel:tn,ab,ti OR quetastad:tn,ab,ti OR queteper:tn,ab,ti OR quetex:tn,ab,ti OR quetiafair:tn,ab,ti OR quetialan:tn,ab,ti OR quetiamylan:tn,ab,ti OR quetiazic:tn,ab,ti OR quetifi:tn,ab,ti OR quetilis:tn,ab,ti OR quetin:tn,ab,ti OR quetiratio:tn,ab,ti OR quetiron:tn,ab,ti OR quetiser:tn,ab,ti OR quetkare:tn,ab,ti OR quetrop:tn,ab,ti OR qurax:tn,ab,ti OR qutipin:tn,ab,ti OR “q win”:tn,ab,ti OR raikar:tn,ab,ti OR resirentin:tn,ab,ti OR rocoz:tn,ab,ti OR seotiapim:tn,ab,ti OR sequa:tn,ab,ti OR sequase:tn,ab,ti OR seratio:tn,ab,ti OR seresano:tn,ab,ti OR serex:tn,ab,ti OR seronia:tn,ab,ti OR seropia:tn,ab,ti OR seroquin:tn,ab,ti OR setinin:tn,ab,ti OR “shu si”:tn,ab,ti OR sondate:tn,ab,ti OR stadaquel:tn,ab,ti OR symquel:tn,ab,ti OR syquet:tn,ab,ti OR taiqutabs:tn,ab,ti OR tevaquel:tn,ab,ti OR tiaquin:tn,ab,ti OR “tim asf”:tn,ab,ti OR viketo:tn,ab,ti OR vorta:tn,ab,ti OR xeroquel:tn,ab,ti OR norquetiapine:tn,ab,ti

#40 #38 OR #39

#41 #37 AND #40

#42 #32 AND #41

*Central*

#1 MeSH descriptor: [Bipolar Disorder] explode all trees

#2 MeSH descriptor: [Mood Disorders] explode all trees

#3 (Bipolar or “bi polar”)

#4 ((rapid or ultradian) near/5 cycl*)

#5 (cyclothymic* or hypomani* or mania* or manic* or mixed episode* or RCBD)

#6 ((affective* or mood) near/1 (disorder* or disturbance* or dysfunction* or illness* or swing*))

#7 [#1-#6]

#8 MeSH descriptor: [Dibenzothiazepines] explode all trees

#9 (quetiapine or Seroquel or “ICI 204636” or “ICI 204646” or ICI204636 or ICI204646 or Socalm or Tienapine or Alzen or Asicot or Bonogren or Cedrina or Cizyapine or Derin or Equepin or Equeta or Etiagen or Etipin or Geldoren or Generiapin or Gentiapin or Gofyl or Gyrex or Hedonin or Ilufren or Kefrenex or Ketidose or Ketilept or Ketinel or Ketipinor or Ketiron or Ketrel or Ketya or Kventiax or Kwetaplex or Kwetinor or Lantiapin or Lobarr or Loplive or Loquen or Mylaquel or Nantarid or Neurorace or Neutapin or Notiabolfen or Pinapaz or Poetra or Psicotric or QiWei or Qudix or Quelan or Quelor or Quemed or Quemyl or Quentapil or Quentiax or Quepin or Quepita or Quepsan or Questax or Quetapel or Quetastad or Queteper or Quetex or Quetiafair or Quetialan or Quetiamylan or Quetiazic or Quetifi or Quetilis or Quetin or Quetiratio or Quetiron or Quetiser or Quetkare or Quetrop or Qurax or Qutipin or Q-win or Raikar or Resirentin or Rocoz or Seotiapim or Sequa or Sequase or Seratio or Seresano or Serex or Seronia or Seropia or Seroquin or Setinin or “Shu Si” or Sondate or Stadaquel or Symquel or Syquet or Taiqutabs or Tevaquel or Tiaquin or “Tim ASF” or Viketo or Vorta or Xeroquel or norquetiapine)

#10 #8 or #9

#11 #7 and #10

*PsycINFO (Ebsco)*

#1 DE “Affective Disorders” OR DE “Bipolar Disorder” OR DE “Cyclothymic Personality” OR DE “Mania” OR DE “Hypomania”

#2 TI (Bipolar OR “bi polar” OR hypomani* OR mania* OR manic* OR mixed episode* OR RCBD OR Cyclophren* OR cyclothymi* OR mixed depression* OR “maniodepressive” OR “mano depressive”) OR AB (Bipolar OR “bi polar” OR hypomani* OR mania* OR manic* OR mixed episode* OR RCBD OR Cyclophren* OR cyclothymi* OR mixed depression* OR “maniodepressive” OR “mano depressive”) OR TM (Bipolar OR “bi polar” OR hypomani* OR mania* OR manic* OR mixed episode* OR RCBD OR Cyclophren* OR cyclothymi* OR mixed depression* OR “maniodepressive” OR “mano depressive”)

#3 TI ((rapid OR ultradian) N5 cycl*) OR AB ((rapid OR ultradian) N5 cycl*) OR TM ((rapid OR ultradian) AND cycl*)

#4 TI ((affective* OR mood) N1 (disorder* OR disturbance* OR dysfunction* OR illness* OR swing*)) OR AB ((affective* OR mood) N1 (disorder* OR disturbance* OR dysfunction* OR illness* OR swing*)) OR TM ((affective* OR mood) N1 (disorder* OR disturbance* OR dysfunction* OR illness* OR swing*))

#5 S1 OR S2 OR S3 OR S4

#6 DE “Quetiapine”

#7 TX (quetiapine OR Seroquel OR “ICI 204636” OR “ICI 204646” OR ICI204636 OR ICI204646 OR Socalm OR Tienapine OR Alzen OR Asicot OR Bonogren OR Cedrina OR Cizyapine OR Derin OR Equepin OR Equeta OR Etiagen OR Etipin OR Geldoren OR Generiapin OR Gentiapin OR Gofyl OR Gyrex OR Hedonin OR Ilufren OR Kefrenex OR Ketidose OR Ketilept OR Ketinel OR Ketipinor OR Ketiron OR Ketrel OR Ketya OR Kventiax OR Kwetaplex OR Kwetinor OR Lantiapin OR Lobarr OR Loplive OR Loquen OR Mylaquel OR Nantarid OR Neurorace OR Neutapin OR Notiabolfen OR Pinapaz OR Poetra OR Psicotric OR QiWei OR Qudix OR Quelan OR Quelor OR Quemed OR Quemyl OR Quentapil OR Quentiax OR Quepin OR Quepita OR Quepsan OR Questax OR Quetapel OR Quetastad OR Queteper OR Quetex OR Quetiafair OR Quetialan OR Quetiamylan OR Quetiazic OR Quetifi OR Quetilis OR Quetin OR Quetiratio OR Quetiron OR Quetiser OR Quetkare OR Quetrop OR Qurax OR Qutipin OR Q-win OR Raikar OR Resirentin OR Rocoz OR Seotiapim OR Sequa OR Sequase OR Seratio OR Seresano OR Serex OR Seronia OR Seropia OR Seroquin OR Setinin OR “Shu Si” OR Sondate OR Stadaquel OR Symquel OR Syquet OR Taiqutabs OR Tevaquel OR Tiaquin OR “Tim ASF” OR Viketo OR Vorta OR Xeroquel OR norquetiapine)

#8 S6 OR S7

#9 S5 AND S8

*CINAHL (Ebsco)*

#1 (MH “Bipolar Disorder+“) OR (MH “Affective Disorders”) OR (MH “Affective Disorders, Psychotic”)

#2 TI (Bipolar OR “bi polar” OR hypomani* OR mania* OR manic* OR mixed episode* OR RCBD OR Cyclophren* OR cyclothymi* OR mixed depression* OR “maniodepressive” OR “mano depressive”) OR AB (Bipolar OR “bi polar” OR hypomani* OR mania* OR manic* OR mixed episode* OR RCBD OR Cyclophren* OR cyclothymi* OR mixed depression* OR “maniodepressive” OR “mano depressive”)

#3 TI ((rapid OR ultradian) N5 cycl*) OR AB ((rapid OR ultradian) N5 cycl*)

#4 TI ((affective* OR mood) N1 (disorder* OR disturbance* OR dysfunction* OR illness* OR swing*)) OR AB ((affective* OR mood) N1 (disorder* OR disturbance* OR dysfunction* OR illness* OR swing*))

#5 S1 OR S2 OR S3 OR S4

#6 (MH “Quetiapine”)

#7 TX (quetiapine OR Seroquel OR “ICI 204636” OR “ICI 204646” OR ICI204636 OR ICI204646 OR Socalm OR Tienapine OR Alzen OR Asicot OR Bonogren OR Cedrina OR Cizyapine OR Derin OR Equepin OR Equeta OR Etiagen OR Etipin OR Geldoren OR Generiapin OR Gentiapin OR Gofyl OR Gyrex OR Hedonin OR Ilufren OR Kefrenex OR Ketidose OR Ketilept OR Ketinel OR Ketipinor OR Ketiron OR Ketrel OR Ketya OR Kventiax OR Kwetaplex OR Kwetinor OR Lantiapin OR Lobarr OR Loplive OR Loquen OR Mylaquel OR Nantarid OR Neurorace OR Neutapin OR Notiabolfen OR Pinapaz OR Poetra OR Psicotric OR QiWei OR Qudix OR Quelan OR Quelor OR Quemed OR Quemyl OR Quentapil OR Quentiax OR Quepin OR Quepita OR Quepsan OR Questax OR Quetapel OR Quetastad OR Queteper OR Quetex OR Quetiafair OR Quetialan OR Quetiamylan OR Quetiazic OR Quetifi OR Quetilis OR Quetin OR Quetiratio OR Quetiron OR Quetiser OR Quetkare OR Quetrop OR Qurax OR Qutipin OR Q-win OR Raikar OR Resirentin OR Rocoz OR Seotiapim OR Sequa OR Sequase OR Seratio OR Seresano OR Serex OR Seronia OR Seropia OR Seroquin OR Setinin OR “Shu Si” OR Sondate OR Stadaquel OR Symquel OR Syquet OR Taiqutabs OR Tevaquel OR Tiaquin OR “Tim ASF” OR Viketo OR Vorta OR Xeroquel OR norquetiapine)

#8 S6 OR S7

#9 S5 AND S8

*PubMed*

1. ((randomized controlled trial[pt]) OR (controlled clinical trial[pt]) OR (randomised[tiab] OR randomized[tiab]) OR (placebo[tiab]) OR (drug therapy[sh]) OR (randomly[tiab]) OR (trial[tiab]) OR (groups[tiab])) NOT (animals[mh] NOT humans[mh])
2. (Bipolar[tw] OR “bi polar”[tw]) NOT Medline[sb]
3. ((rapid[tw] OR ultradian[tw]) AND cycl*[tw]) NOT Medline[sb]
4. (hypomani*[tw] OR mania*[tw] OR manic*[tw] OR mixed episode*[tw] OR RCBD[tw] OR Cyclophren*[tw] OR cyclothymi*[tw] OR mixed depression*[tw] OR “maniodepressive”[tw] OR “mano depressive”[tw]) NOT Medline[sb]
5. ((affective*[tw] OR mood[tw]) AND (disorder*[tw] OR disturbance*[tw] OR dysfunction*[tw] OR illness*[tw] OR swing*[tw])) NOT Medline[sb]
6. #2 OR #3 OR #4 OR #5
7. quetiapine[tw] OR Seroquel[tw] OR “ICI 204636” OR “ICI 204646”[tw] OR ICI204636[tw] OR ICI204646[tw] OR Socalm[tw] OR Tienapine[tw] OR Alzen[tw] OR Asicot[tw] OR Bonogren[tw] OR Cedrina[tw] OR Cizyapine[tw] OR Derin[tw] OR Equepin[tw] OR Equeta[tw] OR Etiagen[tw] OR Etipin[tw] OR Geldoren[tw] OR Generiapin[tw] OR Gentiapin[tw] OR Gofyl[tw] OR Gyrex[tw] OR Hedonin[tw] OR Ilufren[tw] OR Kefrenex[tw] OR Ketidose[tw] OR Ketilept[tw] OR Ketinel[tw] OR Ketipinor[tw] OR Ketiron[tw] OR Ketrel[tw] OR Ketya[tw] OR Kventiax[tw] OR Kwetaplex[tw] OR Kwetinor[tw] OR Lantiapin[tw] OR Lobarr[tw] OR Loplive[tw] OR Loquen[tw] OR Mylaquel OR Nantarid[tw] OR Neurorace[tw] OR Neutapin[tw] OR Notiabolfen[tw] OR Pinapaz[tw] OR Poetra[tw] OR Psicotric[tw] OR QiWei[tw] OR Qudix[tw] OR Quelan[tw] OR Quelor[tw] OR Quemed[tw] OR Quemyl[tw] OR Quentapil[tw] OR Quentiax[tw] OR Quepin[tw] OR Quepita OR Quepsan[tw] OR Questax[tw] OR Quetapel[tw] OR Quetastad[tw] OR Queteper[tw] OR Quetex[tw] OR Quetiafair[tw] OR Quetialan[tw] OR Quetiamylan[tw] OR Quetiazic[tw] OR Quetifi[tw] OR Quetilis[tw] OR Quetin[tw] OR Quetiratio[tw] OR Quetiron[tw] OR Quetiser[tw] OR Quetkare[tw] OR Quetrop[tw] OR Qurax[tw] OR Qutipin[tw] OR Q-win[tw] OR Raikar[tw] OR Resirentin[tw] OR Rocoz[tw] OR Seotiapim[tw] OR Sequa[tw] OR Sequase[tw] OR Seratio[tw] OR Seresano[tw] OR Serex[tw] OR Seronia[tw] OR Seropia[tw] OR Seroquin[tw] OR Setinin[tw] OR “Shu Si”[tw] OR Sondate[tw] OR Stadaquel[tw] OR Symquel[tw] OR Syquet[tw] OR Taiqutabs[tw] OR Tevaquel[tw] OR Tiaquin[tw] OR “Tim ASF”[tw] OR Viketo[tw] OR Vorta[tw] OR Xeroquel[tw] OR norquetiapine[tw]
8. #6 AND #7
9. #1 AND #8

### Searching trial registries

We will conduct a basic search of ClinicaTrials.gov using the terms below. We will conduct a standard search of the ICTRP Search Portal using the same terms, and we will remove the records from ClinicaTrials.gov from these results before screening.

“Dibenzothiazepines OR quetiapine OR Seroquel OR “ICI 204636” OR “ICI 204646” OR ICI204636 OR ICI204646 OR Socalm OR Tienapine OR Alzen OR Asicot OR Bonogren OR Cedrina OR Cizyapine OR Derin OR Equepin OR Equeta OR Etiagen OR Etipin OR Geldoren OR Generiapin OR Gentiapin OR Gofyl OR Gyrex OR Hedonin OR Ilufren OR Kefrenex OR Ketidose OR Ketilept OR Ketinel OR Ketipinor OR Ketiron OR Ketrel OR Ketya OR Kventiax OR Kwetaplex OR Kwetinor OR Lantiapin OR Lobarr OR Loplive OR Loquen OR Mylaquel OR Nantarid OR Neurorace OR Neutapin OR Notiabolfen OR Pinapaz OR Poetra OR Psicotric OR QiWei OR Qudix OR Quelan OR Quelor OR Quemed OR Quemyl OR Quentapil OR Quentiax OR Quepin OR Quepita OR Quepsan OR Questax OR Quetapel OR Quetastad OR Queteper OR Quetex OR Quetiafair OR Quetialan OR Quetiamylan OR Quetiazic OR Quetifi OR Quetilis OR Quetin OR Quetiratio OR Quetiron OR Quetiser OR Quetkare OR Quetrop OR Qurax OR Qutipin OR Q-win OR Raikar OR Resirentin OR Rocoz OR Seotiapim OR Sequa OR Sequase OR Seratio OR Seresano OR Serex OR Seronia OR Seropia OR Seroquin OR Setinin OR “Shu Si” OR Sondate OR Stadaquel OR Symquel OR Syquet OR Taiqutabs OR Tevaquel OR Tiaquin OR “Tim ASF” OR Viketo OR Vorta OR Xeroquel OR norquetiapine”

## Abbreviations

ANC: absolute neutrophil count
CENTRAL: Cochrane central register of controlled trials
CGIC or CGI: clinician global impression of change
COSTART: coding symbols for thesaurus of adverse reaction terms
CSRs: clinical study reports
DIDA: drug industry document archive
EMA: European Medicines Agency
FDA: Food and Drug Administration
HAM-D: Hamilton rating scale for depression
HIPAA: Health Insurance Portability and Accountability Act
HLGT: high-level group term
HLT: high-level term
ICTRP: International Clinical Trials Registry Platform Search Portal
IMMPACT: initiative on methods, measurement, and pain assessment in clinical trials
IOM: Institute of Medicine
IPD: individual participant data
LLT: lowest level term
MADRS: Montgomery-Åsberg Depression Rating Scale
MedDRA: medical dictionary for regulatory activities
MHRA: Medicines and Healthcare products Regulatory Agency
MICE: multiple imputation by chained equations
MSAS: modified Simpson-Angus scale
PCORI: Patient-Centered Outcomes Research Institute
PMDA: Pharmaceuticals and Medical Devices Agency
PT: preferred term
QOL: quality of life
SOC: system organ class
SRDR: Systematic Review Data Repository
TGA: Therapeutic Goods Administration
WMD: weighted mean difference
YMRS: young mania rating scale
YODA: Yale University Open Data Access
