# Correction to: Integrating multiple data sources (MUDS) for meta-analysis to improve patient-centered outcomes research: a protocol for a systematic review

**DOI:** 10.1186/s13195-018-0351-5

**Published:** 2018-02-16

**Authors:** Evan Mayo-Wilson, Susan Hutfless, Tianjing Li, Gillian Gresham, Nicole Fusco, Jeffrey Ehmsen, James Heyward, Swaroop Vedula, Diana Lock, Jennifer Haythornthwaite, Jennifer L. Payne, Theresa Cowley, Elizabeth Tolbert, Lori Rosman, Claire Twose, Elizabeth A. Stuart, Hwanhee Hong, Peter Doshi, Catalina Suarez-Cuervo, Sonal Singh, Kay Dickersin

**Affiliations:** 1 Center for Clinical Trials and Evidence Synthesis, Department of Epidemiology, Johns Hopkins Bloomberg School of Public Health, 615 North Wolfe Street, Baltimore, MD 21205 USA; 2 Department of Gastroenterology and Hepatology, Johns Hopkins School of Medicine, 600 North Wolfe Street, Blalock 449, Baltimore, MD 21287 USA; 3 Department of Neurology, Johns Hopkins School of Medicine, 855 North Wolfe Street, Baltimore, MD 21205 USA; 4 Laboratory for Computational Sensing and Robotics, Johns Hopkins Whiting School of Engineering, 3400 North Charles St, Baltimore, MD 21218 USA; 5 Department of Health Policy and Management, Johns Hopkins Bloomberg School of Public Health, 615 North Wolfe Street, Baltimore, MD 21205 USA; 6 Behavioral Biology, Center for Mind-Body Research, Johns Hopkins School of Medicine, 5501 Hopkins Bayview Circle, Baltimore, MD 21224 USA; 7 Psychiatry and Behavioral Sciences, Johns Hopkins School of Medicine, 500 North Broadway, Suite 305, Baltimore, MD 21205 USA; 8 The TMJ Association, Ltd, P.O. Box 26770, Milwaukee, WI 53226-0770 USA; 9 Peabody Institute, Johns Hopkins University, 1 East Mount Vernon Place, Baltimore, MD 21202 USA; 10 Welch Medical Library, Johns Hopkins School of Medicine, 615 North Wolfe Street, Baltimore, MD 21205 USA; 11 Department of Mental Health, Johns Hopkins Bloomberg School of Public Health, 624, North Broadway, Baltimore, MD 21205 USA; 12 University of Maryland School of Pharmacy, 220 Arch Street, Baltimore, MD 21201 USA; 13 Center for Public Health and Human Rights, Johns Hopkins School of Medicine, 615 N Wolfe, St, Baltimore, MD 21205 USA

## Correction

The correct title of the article [1] should be “Integrating multiple data sources (MUDS) for meta-analysis to improve patient-centered outcomes research: a protocol”. The article is a protocol for a methodological study, not a systematic review.
